# Supplementary material for: How valuable are the questions and answers generated by large language models in oral and maxillofacial surgery?
Source: PLoS One. 2025 May 28;20(5):e0322529. doi: 10.1371/journal.pone.0322529 (PMC12119001; doi:10.1371/journal.pone.0322529)
Supplement: S1 File — (ZIP) [file pone.0322529.s001.zip › claude 3/CO - CO.docx]

1. Which of the following is the most common site for oral cancer?

a) Buccal mucosa

b) Floor of the mouth

c) Hard palate

d) Tongue

e) Gingiva

Answer: d) Tongue

Explanation: The tongue is the most common site for oral cancer due to its high exposure to carcinogens from smoking and alcohol consumption, as well as its rich blood supply and lymphatic drainage. The lateral borders and ventral surface of the tongue are particularly susceptible.

2. A 45-year-old male presents with a painless, firm, and fixed lymph node in the submandibular region. What is the most likely diagnosis?

a) Reactive lymphadenitis

b) Sialadenitis

c) Lymphoma

d) Metastatic oral cancer

e) Lipoma

Answer: d) Metastatic oral cancer

Explanation: A painless, firm, and fixed lymph node in the submandibular region in a middle-aged male is highly suspicious for metastatic oral cancer. The characteristics of being painless, firm, and fixed are typical of malignant lymph node involvement, and the submandibular region is a common site for metastasis from oral cancers.

3. Which of the following is the most common type of odontogenic tumor?

a) Ameloblastoma

b) Odontoma

c) Odontogenic myxoma

d) Adenomatoid odontogenic tumor

e) Calcifying epithelial odontogenic tumor

Answer: b) Odontoma

Explanation: Odontomas are the most common type of odontogenic tumor. They are considered developmental anomalies (hamartomas) rather than true neoplasms. Odontomas are composed of normal dental tissues that are abnormally arranged, and they are often discovered incidentally on routine dental radiographs.

4. A patient presents with a unilateral, painful swelling in the preauricular region that is exacerbated by eating. What is the most likely diagnosis?

a) Mumps

b) Sialolithiasis

c) Sjögren's syndrome

d) Parotid gland tumor

e) Temporomandibular joint disorder

Answer: b) Sialolithiasis

Explanation: The symptoms described - unilateral, painful swelling in the preauricular region that worsens with eating - are classic signs of sialolithiasis (salivary stones) affecting the parotid gland. The pain and swelling typically increase during meals due to obstruction of salivary flow by the stone when salivation increases.

5. Which of the following is the most common site for maxillofacial fractures?

a) Nasal bones

b) Zygoma

c) Mandible

d) Orbital floor

e) Maxilla

Answer: c) Mandible

Explanation: The mandible is the most common site for maxillofacial fractures. This is due to its prominence, mobility, and horseshoe shape, which makes it vulnerable to fractures at several points. Common causes include motor vehicle accidents, interpersonal violence, and falls.

6. A patient presents with a painless, slow-growing mass in the parotid gland. Which of the following is the most likely diagnosis?

Answer: a) Pleomorphic adenoma

Explanation: Pleomorphic adenoma is the most common benign tumor of the parotid gland. It typically presents as a painless, slow-growing mass, which aligns with the description in the question.

7. Which of the following nerves is most commonly injured during third molar extraction?

Answer: d) Inferior alveolar nerve

Explanation: The inferior alveolar nerve runs through the mandibular canal and is in close proximity to the roots of the third molars, making it the most susceptible to injury during extraction.

8. A patient presents with a unilateral, painful, erythematous swelling in the submandibular region. Which of the following is the most likely diagnosis?

Answer: b) Submandibular sialadenitis

Explanation: The symptoms described are typical of acute sialadenitis, an infection of the salivary gland. The submandibular gland is commonly affected due to its anatomy and slower salivary flow.

9. Which of the following is the most common type of ameloblastoma?

Answer: b) Solid/multicystic ameloblastoma

Explanation: Solid/multicystic ameloblastoma is the most common variant, accounting for about 80% of all ameloblastomas. It tends to be more aggressive and has a higher recurrence rate compared to other types.

10. A patient presents with a painful, non-healing ulcer on the lateral border of the tongue that has been present for 6 weeks. Which of the following is the most appropriate next step in management?

Answer: b) Perform an incisional biopsy

Explanation: Any non-healing ulcer present for more than 2-3 weeks, especially on a high-risk site like the lateral border of the tongue, should be biopsied to rule out oral cancer.

11. Which of the following is the most common site for osteonecrosis of the jaw associated with bisphosphonate therapy?

Answer: b) Mandible

Explanation: The mandible, particularly the posterior region, is most commonly affected by bisphosphonate-related osteonecrosis due to its relatively poor blood supply and high remodeling rate.

12. A patient presents with a rapidly growing, painful swelling in the mandible. Radiographic examination reveals a multilocular radiolucency with irregular borders. Which of the following is the most likely diagnosis?

Answer: e) Osteosarcoma

Explanation: The rapid growth, pain, and aggressive radiographic appearance are suggestive of a malignant bone tumor, with osteosarcoma being the most likely in this scenario.

13. Which of the following is the most common type of maxillofacial trauma in pediatric patients?

Answer: c) Nasal fractures

Explanation: Due to the prominence of the nose and its relatively weak structure, nasal fractures are the most common facial fractures in children.

14. A patient presents with a painless, slow-growing mass in the submandibular gland. Fine needle aspiration biopsy reveals a mixture of epithelial and myoepithelial cells. Which of the following is the most likely diagnosis?

Answer: a) Pleomorphic adenoma

Explanation: The clinical presentation and cytological findings are typical of pleomorphic adenoma, which can occur in the submandibular gland, although less commonly than in the parotid.

15. Which of the following is the most common site for cervical lymph node metastasis in oral squamous cell carcinoma?

Answer: b) Level II (upper jugular nodes)

Explanation: Level II nodes are the most common site for metastasis from oral cavity cancers due to the lymphatic drainage patterns of the oral cavity.

16. A patient presents with a unilateral, diffuse swelling of the parotid gland associated with dry mouth and dry eyes. Which of the following is the most likely diagnosis?

Answer: a) Sjögren's syndrome

Explanation: The combination of salivary gland swelling with dry mouth and eyes is characteristic of Sjögren's syndrome, an autoimmune condition affecting exocrine glands.

17. Which of the following is the most common type of odontogenic cyst?

Answer: a) Radicular cyst

Explanation: Radicular cysts, associated with non-vital teeth, are the most common odontogenic cysts, arising from inflammatory stimulation of epithelial cell rests of Malassez.

18. A patient presents with a firm, painless mass in the floor of the mouth that causes elevation of the tongue. Which of the following is the most likely diagnosis?

Answer: b) Dermoid cyst

Explanation: The description is typical of a dermoid cyst in the floor of the mouth, which often presents as a firm, painless mass that can cause tongue elevation.

19. Which of the following is the most common site for osteoradionecrosis of the jaws?

Answer: b) Mandible

Explanation: The mandible, due to its denser bone and more tenuous blood supply, is more susceptible to osteoradionecrosis than the maxilla.

20. A patient presents with a unilateral, fluctuant swelling in the buccal vestibule adjacent to a vital tooth. Radiographic examination reveals no abnormalities. Which of the following is the most likely diagnosis?

Answer: c) Nasolabial cyst

Explanation: The location and clinical presentation, along with the lack of radiographic findings, are consistent with a nasolabial cyst, which is a non-odontogenic, soft tissue cyst.

21. Which of the following is the most common site for a lingual thyroid?

Answer: b) Posterior tongue

Explanation: Lingual thyroid, when present, is most commonly found at the base of the tongue, near the foramen cecum.

22. A patient presents with a rapidly growing, painless mass in the parotid gland. Fine needle aspiration biopsy reveals a predominance of lymphoid cells. Which of the following is the most likely diagnosis?

Answer: b) Warthin's tumor

Explanation: The clinical presentation and cytological findings of predominant lymphoid cells are characteristic of Warthin's tumor, also known as papillary cystadenoma lymphomatosum.

23. Which of the following is the most common type of maxillofacial fracture in elderly patients?

Answer: a) Mandibular fractures

Explanation: In the elderly, mandibular fractures are most common due to the prominence of the mandible and the higher likelihood of falls in this population.

24. A patient presents with a slow-growing, painless mass in the submandibular gland. Fine needle aspiration biopsy reveals a predominance of uniform, basaloid cells with a cribriform pattern. Which of the following is the most likely diagnosis?

Answer: d) Adenoid cystic carcinoma

Explanation: The cytological findings of basaloid cells in a cribriform pattern are highly suggestive of adenoid cystic carcinoma, a malignant salivary gland tumor.

25. Which of the following is the most common site for a dentigerous cyst?

Answer: d) Mandibular posterior region

Explanation: Dentigerous cysts most commonly occur in association with impacted mandibular third molars in the posterior mandible.

26. A patient presents with a diffuse, bilateral swelling of the parotid glands associated with fever, malaise, and myalgia. Which of the following is the most likely diagnosis?

Answer: e) Mumps

Explanation: The bilateral parotid swelling accompanied by systemic symptoms is characteristic of mumps, a viral infection caused by the mumps virus.

27. Which of the following is the most common type of odontogenic tumor in children?

Answer: b) Odontoma

Explanation: Odontomas are the most frequently encountered odontogenic tumors in children, often discovered incidentally on routine dental radiographs.

28. A patient presents with a painful, non-healing ulcer on the ventral surface of the tongue that has been present for 3 months. The patient has a history of heavy smoking and alcohol consumption. Which of the following is the most appropriate next step in management?

Answer: b) Perform an incisional biopsy

Explanation: Given the duration of the lesion, its location, and the patient's risk factors, an incisional biopsy is crucial to rule out oral squamous cell carcinoma.

29. Which of the following is the most common site for a ranula?

Answer: b) Floor of the mouth

Explanation: Ranulas typically occur in the floor of the mouth, arising from the sublingual gland or its ducts.

30. A patient presents with a rapidly growing, painful mass in the mandible. Radiographic examination reveals a radiolucent lesion with irregular borders and a "sunburst" appearance. Which of the following is the most likely diagnosis?

Answer: e) Osteosarcoma

Explanation: The rapid growth, pain, and characteristic "sunburst" radiographic appearance are highly suggestive of osteosarcoma, a malignant bone tumor.

31. Which of the following is the most common type of maxillofacial trauma in adult patients?

Answer: a) Mandibular fractures

Explanation: In adults, mandibular fractures are the most common type of maxillofacial fracture due to the mandible's prominence and vulnerability to impact.

32. A patient presents with a slow-growing, painless mass in the parotid gland. Fine needle aspiration biopsy reveals a predominance of mucous cells. Which of the following is the most likely diagnosis?

Answer: c) Mucoepidermoid carcinoma

Explanation: The presence of mucous cells on cytology is characteristic of mucoepidermoid carcinoma, the most common malignant salivary gland tumor.

33. Which of the following is the most common site for a nasopalatine duct cyst?

Answer: a) Maxillary anterior region

Explanation: Nasopalatine duct cysts occur in the anterior maxilla, typically in the midline behind the central incisors.

34. A patient presents with a firm, painless mass in the tongue that has been slowly growing for 6 months. Which of the following is the most likely diagnosis?

Answer: b) Fibroma

Explanation: A slow-growing, firm, painless mass in the tongue is most likely to be a fibroma, a benign tumor of fibrous connective tissue.

35. Which of the following is the most common type of odontogenic tumor in adults?

Answer: a) Ameloblastoma

Explanation: In adults, ameloblastoma is the most common odontogenic tumor, typically occurring in the posterior mandible.

36. A patient presents with a unilateral, painful swelling in the submandibular region that is exacerbated by eating. Radiographic examination reveals a radiopaque mass within the submandibular duct. Which of the following is the most likely diagnosis?

Answer: a) Submandibular sialolithiasis

Explanation: The clinical presentation and radiographic findings are typical of a salivary stone (sialolith) in the submandibular duct.

37. Which of the following is the most common site for a mucoepidermoid carcinoma?

Answer: a) Parotid gland

Explanation: Mucoepidermoid carcinoma most frequently occurs in the parotid gland, though it can affect other major and minor salivary glands.

38. A patient presents with a painful, non-healing ulcer on the lateral border of the tongue that has been present for 4 weeks. The patient has a history of chewing tobacco. Which of the following is the most appropriate next step in management?

Answer: b) Perform an incisional biopsy

Explanation: Given the duration of the lesion, its location, and the patient's history of tobacco use, a biopsy is necessary to rule out oral squamous cell carcinoma.

39. Which of the following is the most common site for a pleomorphic adenoma?

Answer: a) Parotid gland

Explanation: Pleomorphic adenoma, the most common benign salivary gland tumor, occurs most frequently in the parotid gland.

40. A patient presents with a rapidly growing, painless mass in the posterior mandible. Radiographic examination reveals a multilocular radiolucency with scalloped borders. Which of the following is the most likely diagnosis?

Answer: a) Ameloblastoma

Explanation: The radiographic appearance of a multilocular radiolucency with scalloped borders in the posterior mandible is characteristic of ameloblastoma.

41. Which of the following is the most common type of maxillofacial fracture resulting from motor vehicle accidents?

Answer: e) Le Fort fractures

Explanation: In high-impact traumas like motor vehicle accidents, Le Fort fractures involving the midface are common due to the force of impact on the dashboard or steering wheel.

42. A patient presents with a slow-growing, painless mass in the parotid gland. Fine needle aspiration biopsy reveals a predominance of oncocytic cells. Which of the following is the most likely diagnosis?

Answer: b) Warthin's tumor

Explanation: The presence of oncocytic cells on cytology is characteristic of Warthin's tumor, also known as papillary cystadenoma lymphomatosum.

43. Which of the following is the most common site for a central giant cell granuloma?

Answer: c) Mandibular anterior region

Explanation: Central giant cell granulomas most commonly occur in the anterior mandible, often crossing the midline.

44. A patient presents with a diffuse, bilateral enlargement of the parotid glands associated with xerostomia and rheumatoid arthritis. Which of the following is the most likely diagnosis?

Answer: a) Sjögren's syndrome

Explanation: The combination of salivary gland enlargement, dry mouth, and an associated autoimmune condition (rheumatoid arthritis) is characteristic of Sjögren's syndrome.

45. Which of the following is the most common type of odontogenic cyst associated with an impacted tooth?

Answer: b) Dentigerous cyst

Explanation: Dentigerous cysts are the most common type of developmental odontogenic cyst and are associated with the crown of an unerupted tooth.

46. A patient presents with a painful, non-healing ulcer on the floor of the mouth that has been present for 2 months. The patient has a history of smoking and drinking alcohol. Which of the following is the most appropriate next step in management?

Answer: b) Perform an incisional biopsy

Explanation: Given the duration of the lesion, its location, and the patient's risk factors, an incisional biopsy is crucial to rule out oral squamous cell carcinoma.

47. Which of the following is the most common site for a mucocele?

Answer: e) Lower lip

Explanation: Mucoceles most commonly occur on the lower lip due to the high concentration of minor salivary glands in this area and its susceptibility to trauma.

48. A patient presents with a slow-growing, painless mass in the submandibular gland. Fine needle aspiration biopsy reveals a predominance of clear cells. Which of the following is the most likely diagnosis?

Answer: e) Acinic cell carcinoma

Explanation: The presence of clear cells on cytology from a salivary gland mass is suggestive of acinic cell carcinoma, though it can also occur in other tumors.

49. Which of the following is the most common site for a traumatic bone cyst?

Answer: d) Mandibular posterior region

Explanation: Traumatic bone cysts, also known as simple bone cysts, most commonly occur in the posterior mandible.

50. A patient presents with a rapidly growing, painful mass in the maxillary sinus. Radiographic examination reveals a radiopaque lesion with a "ground glass" appearance. Which of the following is the most likely diagnosis?

Answer: b) Fibrous dysplasia

Explanation: The "ground glass" appearance on radiographs is characteristic of fibrous dysplasia, a benign fibro-osseous lesion that can affect the maxillary sinus.
