# Supplementary material for: How valuable are the questions and answers generated by large language models in oral and maxillofacial surgery?
Source: PLoS One. 2025 May 28;20(5):e0322529. doi: 10.1371/journal.pone.0322529 (PMC12119001; doi:10.1371/journal.pone.0322529)
Supplement: S1 File — (ZIP) [file pone.0322529.s001.zip › claude 3/CO - gpt4 .docx]

Question 1: Which nerve is most commonly injured during a mandibular third molar extraction?

Solution:

1. Consider the anatomical location of the mandibular third molar
2. Identify nerves in close proximity to this area
3. Evaluate the risk of injury for each nerve during extraction

Answer: C. Inferior alveolar nerve

Explanation: The inferior alveolar nerve runs through the mandibular canal, which is often in close proximity to the roots of the third molar. During extraction, this nerve is at the highest risk of injury due to its location.

Question 2: A 45-year-old male presents with a fractured zygomatic arch. Which imaging modality is most suitable to evaluate the fracture?

Solution:

1. Consider the location of the zygomatic arch (part of the facial skeleton)
2. Evaluate different imaging modalities for their ability to visualize bony structures
3. Assess which modality provides the best detail for facial bone fractures

Answer: D. CT scan

Explanation: CT scans provide excellent detail of bony structures and are particularly useful for complex facial fractures. They offer 3D reconstruction capabilities, allowing for better visualization of the fracture's extent and displacement.

Question 3: What is the most common cause of osteonecrosis of the jaw?

Solution:

1. Review potential causes of osteonecrosis
2. Consider frequency of each cause
3. Evaluate recent medical literature on the topic

Answer: B. Bisphosphonate therapy

Explanation: While there are several potential causes of osteonecrosis of the jaw, bisphosphonate therapy has been widely recognized as the most common cause in recent years. These drugs, used to treat osteoporosis and certain cancers, can interfere with bone remodeling and healing.

Question 4: During TMJ surgical procedures, which structure is most at risk of injury?

Solution:

1. Review the anatomy surrounding the temporomandibular joint
2. Identify critical structures in close proximity
3. Consider which structure is most vulnerable during surgical access

Answer: B. Facial nerve

Explanation: The facial nerve passes close to the TMJ. Its branches, particularly the temporal and zygomatic, are at risk during surgical approaches to the TMJ due to their superficial course in this area.

Question 5: What is the first-line surgical intervention for severe obstructive sleep apnea unresponsive to CPAP therapy?

Solution:

1. Consider the pathophysiology of obstructive sleep apnea
2. Review surgical options for addressing upper airway obstruction
3. Evaluate which procedure is most commonly performed as a first surgical step

Answer: C. Uvulopalatopharyngoplasty (UPPP)

Explanation: UPPP is often considered the first-line surgical intervention for severe OSA when CPAP fails. It addresses obstruction at the level of the soft palate and oropharynx, which are common sites of collapse in OSA.

Question 6: What is the typical imaging feature of a dentigerous cyst on a panoramic radiograph?

Solution:

1. Recall the definition and origin of a dentigerous cyst
2. Consider how this would appear on a radiograph
3. Identify the characteristic relationship between the cyst and associated tooth

Answer: B. Radiolucent area surrounding an unerupted tooth

Explanation: A dentigerous cyst characteristically appears as a well-defined radiolucent area surrounding the crown of an unerupted tooth. This is due to its origin from the reduced enamel epithelium of a developing tooth.

Question 7: In the case of a mandibular angle fracture, which of the following is the most crucial factor in deciding the treatment plan?

Solution:

1. Consider various factors affecting fracture management
2. Evaluate which factor most significantly impacts treatment choice
3. Think about how this factor influences the need for surgical intervention

Answer: B. Degree of displacement

Explanation: The degree of displacement is crucial in determining whether a fracture can be managed conservatively or requires surgical intervention. Significantly displaced fractures often necessitate open reduction and internal fixation to ensure proper healing and function.

Question 8: A 25-year-old female presents with a swelling at the angle of the mandible. On imaging, a well-circumscribed, radiolucent lesion with a "soap-bubble" appearance is identified. What is the most likely diagnosis?

Solution:

1. Consider the age and location of the lesion
2. Analyze the radiographic appearance, particularly the "soap-bubble" description
3. Review common odontogenic tumors with this presentation

Answer: A. Ameloblastoma

Explanation: Ameloblastomas typically present as multilocular radiolucent lesions with a "soap-bubble" or "honeycomb" appearance on radiographs. They are often found in the mandibular angle region and are more common in young adults.

Question 9: What surgical technique is typically used to correct mandibular prognathism?

Solution:

1. Consider the goal of correcting mandibular prognathism

2. Review common orthognathic surgical techniques

3. Identify which technique allows for posterior repositioning of the mandible

Answer: B. Bilateral sagittal split osteotomy (BSSO)

Explanation: BSSO is the standard technique for mandibular setback in cases of mandibular prognathism. It allows for precise repositioning of the mandible while maintaining blood supply to the segments.

Question 10: In the setting of facial trauma, which Le Fort fracture type involves separation of the midface from the cranial base?

Solution:

1. Review the classification of Le Fort fractures

2. Identify the fracture line paths for each type

3. Determine which type extends to the cranial base

Answer: C. Le Fort III

Explanation: Le Fort III fracture, also known as craniofacial disjunction, involves separation of the entire midface from the cranial base. The fracture line extends through the nasofrontal suture, medial orbital wall, lateral orbital wall, and zygomatic arch.

Question 11: A 60-year-old patient presents with an oral squamous cell carcinoma involving the floor of the mouth. Which of the following is the most appropriate initial imaging modality for staging the tumor?

Solution:

1. Consider the need for assessing both soft tissue and bony involvement

2. Evaluate the ability of different imaging modalities to visualize these structures

3. Think about the importance of assessing lymph node involvement

Answer: D. Contrast-enhanced CT scan

Explanation: Contrast-enhanced CT scan provides detailed information about the tumor's extent, bony involvement, and lymph node status. It's the initial imaging modality of choice for staging oral squamous cell carcinoma.

Question 12: Which condition is characterized by bilateral enlargement of the parotid glands and is often associated with chronic alcohol abuse?

Solution:

1. Consider conditions affecting the parotid glands

2. Identify those associated with bilateral enlargement

3. Evaluate the link between these conditions and alcohol abuse

Answer: E. Sialadenosis

Explanation: Sialadenosis, also known as sialosis, is a non-inflammatory enlargement of salivary glands, often bilateral. It's frequently associated with chronic alcoholism, as well as other metabolic disorders.

Question 13: What is the preferred surgical treatment for a benign tumor located at the angle of the mandible?

Solution:

1. Consider the goal of treating a benign tumor

2. Evaluate different surgical approaches

3. Determine which approach balances complete removal with preservation of function

Answer: B. Marginal resection

Explanation: Marginal resection allows for complete removal of the tumor while preserving mandibular continuity. This approach is suitable for benign tumors, balancing tumor eradication with functional preservation.

Question 14: A patient presents with a suspected mandibular fracture. Which physical examination finding is most indicative of this diagnosis?

Solution:

1. Consider the biomechanics of mandibular function

2. Review common signs of mandibular fracture

3. Identify which sign most directly indicates disruption of normal mandibular anatomy

Answer: B. Anterior open bite

Explanation: An anterior open bite often indicates a bilateral posterior mandibular fracture (commonly condylar). It occurs due to the pull of the masseter and medial pterygoid muscles on the posterior segment, causing it to rotate upward and backward.

Question 15: Which type of flap is commonly used for large defects in oral cancer reconstruction that require both soft tissue and bony reconstruction?

Solution:

1. Consider flaps that can provide both bone and soft tissue

2. Evaluate donor sites that can provide sufficient bone length

3. Think about the vascularity and reliability of different flaps

Answer: D. Fibula free flap

Explanation: The fibula free flap is ideal for large oral defects requiring both bone and soft tissue. It provides a long segment of bone that can be osteotomized to recreate mandibular contour, along with a reliable skin paddle for soft tissue coverage.

Question 16: Which of these statements about odontogenic keratocyst (OKC) is true?

Solution:

1. Review the characteristics of OKCs

2. Consider their location, behavior, and association with syndromes

3. Evaluate their response to treatment

Answer: B. It has a high recurrence rate after treatment.

Explanation: OKCs are known for their high recurrence rate, which can be up to 30% or higher. This is due to their thin, friable lining which can be difficult to completely remove, and their potential for satellite cysts.

Question 17: In orthognathic surgery, which of the following is considered when planning for a mandibular setback?

Solution:

1. Think about the changes in mandibular position during setback

2. Consider the effect on surrounding structures and overall facial profile

3. Evaluate the impact on the mandibular angle

Answer: B. Increase in mandibular angle

Explanation: During mandibular setback, the mandibular angle typically increases. This is because the posterior border of the mandible is moved backwards, increasing the angle between the mandibular plane and the Frankfort horizontal plane.

Question 18: Which characteristic feature is often seen in panoramic radiographs of patients with amelogenesis imperfecta?

Solution:

1. Consider the primary defect in amelogenesis imperfecta

2. Think about how this defect would appear radiographically

3. Evaluate the impact on overall tooth structure appearance

Answer: B. Generalized thinning of enamel

Explanation: Amelogenesis imperfecta is a hereditary defect of enamel formation. Radiographically, this often appears as generalized thinning of the enamel layer, with reduced contrast between enamel and dentin.

Question 19: A 35-year-old male is involved in a road traffic accident and presents with a midface fracture. The clinical and radiological findings suggest a zygomaticomaxillary complex fracture. Which clinical sign is most likely to be present?

Solution:

1. Review the anatomy of the zygomaticomaxillary complex

2. Consider the common fracture patterns in this area

3. Identify which clinical sign would be most indicative of disruption to this complex

Answer: D. Inferior orbital rim step deformity

Explanation: A step deformity at the inferior orbital rim is a classic sign of a zygomaticomaxillary complex fracture. This occurs due to displacement of the zygoma, which forms part of the inferior orbital rim.

Question 20: What is the main advantage of using intraoperative navigation systems in maxillofacial surgery?

Solution:

1. Consider the challenges in maxillofacial surgery

2. Review the capabilities of navigation systems

3. Evaluate how these systems can address surgical challenges

Answer: D. Increased precision in anatomical localization

Explanation: Intraoperative navigation systems provide real-time, three-dimensional information about the position of surgical instruments relative to the patient's anatomy. This increases precision in anatomical localization, particularly useful in complex reconstructive procedures or when operating near critical structures.

Question 21: Which muscle is most commonly involved in trismus following a mandibular third molar extraction?

Solution:

1. Review the muscles of mastication

2. Consider their anatomical relationship to the third molar

3. Evaluate which muscle is most likely to be affected during the procedure

Answer: C. Medial pterygoid

Explanation: The medial pterygoid muscle is located close to the surgical site of mandibular third molar extraction. Inflammation or trauma to this muscle during the procedure can lead to trismus.

Question 22: Which of the following is a common sign of an orbital floor fracture (blowout fracture)?

Solution:

1. Consider the anatomy of the orbital floor

2. Review the mechanism of blowout fractures

3. Evaluate the impact on orbital contents and eye position

Answer: C. Enophthalmos

Explanation: Enophthalmos (posterior displacement of the globe) is a common sign of orbital floor fractures. It occurs due to an increase in orbital volume as orbital contents herniate into the maxillary sinus through the fractured floor.

Question 23: Which antibiotic is recommended as first-line therapy for odontogenic infections that are anaerobic in nature?

Solution:

1. Consider the typical microbiology of odontogenic infections

2. Review antibiotics effective against anaerobic bacteria

3. Evaluate which antibiotic is most commonly used in dental practice

Answer: D. Metronidazole

Explanation: Metronidazole is highly effective against anaerobic bacteria, which are common in odontogenic infections. It's often used as a first-line therapy, especially when anaerobic involvement is suspected.

Question 24: A panoramic radiograph of a patient reveals a radiopaque lesion with a radiolucent halo in the posterior mandible. What is the most likely diagnosis?

Solution:

1. Consider lesions that appear radiopaque on radiographs

2. Evaluate the significance of the radiolucent halo

3. Think about common odontogenic tumors in this location

Answer: C. Odontoma

Explanation: Odontomas typically appear as well-defined radiopaque lesions surrounded by a thin radiolucent halo. They are common odontogenic tumors often found in the posterior mandible.

Question 25: Which condition is commonly characterized by the presence of painless, bilateral swelling of the preauricular areas?

Solution:

1. Consider conditions affecting the salivary glands

2. Review those that cause bilateral swelling

3. Evaluate which condition is typically painless and chronic

Answer: D. Sjögren's syndrome

Explanation: Sjögren's syndrome is an autoimmune disorder that often presents with painless, bilateral swelling of the parotid glands (located in the preauricular area). This swelling is typically chronic and progressive.

Question 26: A 50-year-old male presents with difficulty swallowing and severe tooth sensitivity. Oral examination reveals erythematous and smooth tongue lesions. What dietary deficiency might be causing these symptoms?

Solution:

1. Consider vitamins and minerals essential for oral health

2. Review deficiencies that can cause glossitis and oral symptoms

3. Evaluate which deficiency is most commonly associated with these specific symptoms

Answer: D. Vitamin B12

Explanation: Vitamin B12 deficiency can cause glossitis (inflammation of the tongue), resulting in a smooth, erythematous tongue. It can also lead to oral paresthesia, burning sensations, and difficulty swallowing.

Question 27: Which of the following is the primary concern in the management of a patient with a dislocated TMJ?

Solution:

1. Consider the immediate effects of TMJ dislocation

2. Review potential complications if left untreated

3. Evaluate the most urgent need for the patient

Answer: A. Reducing the dislocation manually

Explanation: The primary concern in TMJ dislocation is to reduce it promptly. Manual reduction restores normal jaw function, alleviates pain, and prevents potential long-term complications like chronic dislocation or arthritic changes.

Question 28: A 35-year-old patient presents with a jaw swelling associated with an unerupted mandibular third molar. Radiographs show a well-defined radiolucent lesion with a sclerotic border. What is the most likely diagnosis?

Solution:

1. Consider common cystic lesions associated with unerupted teeth

2. Review the radiographic appearance of these lesions

3. Evaluate which lesion most commonly occurs with mandibular third molars

Answer: C. Dentigerous cyst

Explanation: A dentigerous cyst typically appears as a well-defined radiolucent lesion with a sclerotic border, associated with the crown of an unerupted tooth. They are commonly found with mandibular third molars.

Question 29: Which of the following is least likely to be associated with osteoradionecrosis of the jaw?

Solution:

1. Review the pathophysiology of osteoradionecrosis

2. Consider factors that contribute to its development

3. Evaluate which option is not directly related to radiation effects on bone

Answer: D. Chemotherapy

Explanation: While chemotherapy can have oral complications, it is not directly associated with osteoradionecrosis. Osteoradionecrosis is specifically related to radiation therapy, which impairs the bone's ability to heal and remodel.

Question 30: Which nerve block is most commonly used for pain relief during maxillary anterior teeth procedures?

Solution:

1. Review the innervation of maxillary anterior teeth

2. Consider the different nerve blocks used in the maxilla

3. Evaluate which block most efficiently anesthetizes this region

Answer: E. Anterior superior alveolar nerve block

Explanation: The anterior superior alveolar nerve block effectively anesthetizes the maxillary anterior teeth. This block targets the anterior superior alveolar nerve, a branch of the infraorbital nerve.

Question 31: What is the most common type of malignant tumor found within the salivary glands?

Solution:

1. Review common salivary gland malignancies

2. Consider their relative frequencies

3. Evaluate which is most prevalent across all salivary glands

Answer: C. Mucoepidermoid carcinoma

Explanation: Mucoepidermoid carcinoma is the most common malignant tumor of the salivary glands overall. It can occur in both major and minor salivary glands, with the parotid gland being the most common site.

Question 32: In the management of mandibular fractures, which of the following is considered the gold standard for rigid fixation?

Solution:

1. Consider the goals of fracture fixation

2. Review different fixation methods

3. Evaluate which method provides the most stable fixation

Answer: C. Non-resorbable plates and screws

Explanation: Non-resorbable plates and screws are considered the gold standard for rigid fixation of mandibular fractures. They provide stable fixation that allows for immediate function and promotes primary bone healing.

Question 33: Which condition is characterized by "onion skin" periosteal reaction on radiographs?

Solution:

1. Consider conditions that cause periosteal reaction

2. Review the appearance of different types of periosteal reactions

3. Identify which condition specifically causes the "onion skin" pattern

Answer: B. Ewing's sarcoma

Explanation: The "onion skin" or lamellated periosteal reaction is characteristic of Ewing's sarcoma. This appearance is due to layers of new bone formation in response to the rapidly growing tumor.

Question 34: A 40-year-old female presents with periodic episodes of jaw pain, clicking, and limited mouth opening. What is the most likely diagnosis?

Solution:

1. Consider common temporomandibular joint disorders

2. Review the symptoms associated with each

3. Evaluate which condition best matches the described symptoms

Answer: D. Internal derangement of the TMJ

Explanation: Internal derangement of the TMJ, often involving disc displacement, typically presents with episodes of pain, clicking (as the disc moves in and out of position), and limited mouth opening.

Question 35: Which of the following is a hallmark feature of fibrous dysplasia in the maxillofacial region?

Solution:

1. Consider the pathophysiology of fibrous dysplasia

2. Review its effects on bone structure

3. Evaluate how these changes appear radiographically

Answer: B. Ground-glass appearance on radiographs

Explanation: Fibrous dysplasia characteristically shows a "ground-glass" appearance on radiographs. This is due to the replacement of normal bone with fibrous tissue and immature woven bone.

Question 36: A 30-year-old male presents with chronic non-healing ulcers on the oral mucosa. A biopsy reveals granulomas with multinucleated giant cells. What is the most likely diagnosis?

Solution:

1. Consider conditions that cause oral ulceration

2. Review diseases associated with granulomatous inflammation

3. Evaluate which condition commonly affects the oral cavity

Answer: B. Sarcoidosis

Explanation: Sarcoidosis is a multisystem granulomatous disorder that can affect the oral cavity. The presence of non-caseating granulomas with multinucleated giant cells is characteristic of this condition.

Question 37: A patient with a history of bisphosphonate use presents with jaw pain and exposed bone. What is the likely diagnosis?

Solution:

1. Consider complications associated with bisphosphonate therapy

2. Review the signs and symptoms of these complications

3. Evaluate which condition matches the presented symptoms

Answer: B. Osteonecrosis of the jaw

Explanation: Bisphosphonate-related osteonecrosis of the jaw (BRONJ) is characterized by exposed, necrotic bone in the maxillofacial region in patients with a history of bisphosphonate therapy.

Question 38: A child presents with dental trauma resulting in the avulsion of a primary incisor. What is the recommended management?

Solution:

1. Consider the differences in managing primary vs. permanent tooth avulsion

2. Review potential impacts on the developing permanent tooth

3. Evaluate the risk-benefit ratio of different management options

Answer: C. Do not replant the tooth and schedule follow-up

Explanation: Primary teeth should not be replanted after avulsion

Certainly, I'll continue with the remaining questions:

Question 39: In which syndrome is there a triad of cleft lip/palate, mandibular hypoplasia, and hypoplastic zygomas?

Solution:

1. Review craniofacial syndromes

2. Consider those affecting the first and second branchial arches

3. Identify which syndrome specifically affects these three areas

Answer: B. Treacher Collins syndrome

Explanation: Treacher Collins syndrome is characterized by this triad of craniofacial abnormalities due to abnormal development of the first and second branchial arches.

Question 40: Which type of cyst is most commonly associated with non-vital teeth?

Solution:

1. Consider types of odontogenic cysts

2. Review their etiology

3. Evaluate which is directly related to pulpal necrosis

Answer: A. Radicular cyst

Explanation: Radicular cysts, also known as periapical cysts, are the most common cystic lesions associated with non-vital teeth. They develop from epithelial cell rests of Malassez in response to inflammation from pulpal necrosis.

Question 41: Which factor is most predictive of the likelihood of a patient developing post-operative infections after orthognathic surgery?

Solution:

1. Consider potential risk factors for post-operative infections

2. Review the impact of oral health on surgical outcomes

3. Evaluate which factor most directly affects the oral bacterial load

Answer: D. Pre-existing periodontal disease

Explanation: Pre-existing periodontal disease significantly increases the risk of post-operative infections in orthognathic surgery due to the higher bacterial load in the oral cavity.

Question 42: A 25-year-old patient complains of pain, swelling, and limited mouth opening a few days after a lower third molar extraction. What is the most likely diagnosis?

Solution:

1. Consider common complications of third molar extraction

2. Review the timing and symptoms of these complications

3. Evaluate which complication matches the presented symptoms

Answer: C. Submasseteric abscess

Explanation: A submasseteric abscess can develop as a complication of lower third molar extraction. It presents with pain, swelling, and trismus (limited mouth opening) a few days after the procedure.

Question 43: Which syndrome is characterized by bilateral cleft lip and palate, absent upper central incisors, and mesiodens?

Solution:

1. Review syndromes associated with cleft lip and palate

2. Consider those with specific dental anomalies

3. Identify which syndrome includes these specific features

Answer: E. Ellis-van Creveld syndrome

Explanation: Ellis-van Creveld syndrome is a rare genetic disorder characterized by short limbs, polydactyly, ectodermal dysplasia, and the specific oral features mentioned, including bilateral cleft lip and palate, missing upper incisors, and supernumerary teeth (mesiodens).

Question 44: A 50-year-old male presents with a painless, slow-growing mass in the hard palate. Biopsy shows pleomorphic adenoma. What is the appropriate management?

Solution:

1. Consider the nature of pleomorphic adenomas

2. Review treatment options for salivary gland tumors

3. Evaluate the most effective approach for complete removal

Answer: B. Surgical excision with clear margins

Explanation: Pleomorphic adenomas are benign but can recur if not completely excised. The standard treatment is surgical excision with clear margins to ensure complete removal and minimize the risk of recurrence.

Question 45: Which imaging technique is most appropriate for evaluating the soft tissue extent of oral cancers?

Solution:

1. Consider the need for soft tissue detail in cancer staging

2. Review capabilities of different imaging modalities

3. Evaluate which provides the best soft tissue contrast

Answer: E. MRI

Explanation: MRI provides excellent soft tissue contrast and is superior for evaluating the extent of soft tissue involvement in oral cancers. It's particularly useful for assessing perineural spread and deep tissue invasion.

Question 46: What is the primary reason for immobilizing the mandible in patients with condylar fractures?

Solution:

1. Consider the biomechanics of the mandible

2. Review the healing process of condylar fractures

3. Evaluate the impact of movement on fracture alignment

Answer: B. To ensure proper alignment of the condyle

Explanation: Immobilization helps maintain proper alignment of the condyle during the healing process. This is crucial for restoring normal jaw function and preventing complications like malocclusion or TMJ disorders.

Question 47: A 55-year-old male presents with paresthesia of the lower lip and chin following a dental extraction. Which nerve is most likely affected?

Solution:

1. Review the innervation of the lower lip and chin

2. Consider which nerves are at risk during dental extractions

3. Identify which nerve specifically supplies sensation to this area

Answer: C. Mental nerve

Explanation: The mental nerve, a terminal branch of the inferior alveolar nerve, provides sensory innervation to the lower lip and chin. Damage to this nerve during lower tooth extraction can cause paresthesia in these areas.

Question 48: Which condition is diagnosed by the presence of linearly arranged multinucleated giant cells and fibrous stroma on a biopsy of a jaw lesion?

Solution:

1. Consider lesions characterized by giant cells

2. Review the histological features of these lesions

3. Identify which specifically shows linearly arranged giant cells

Answer: C. Central giant cell granuloma

Explanation: Central giant cell granuloma is characterized histologically by the presence of multinucleated giant cells arranged in a linear fashion within a fibrous stroma.

Question 49: In patients with advanced osteoradionecrosis of the mandible, what is the most definitive treatment?

Solution:

1. Consider the pathophysiology of osteoradionecrosis

2. Review conservative and surgical treatment options

3. Evaluate which approach addresses both the necrotic bone and soft tissue defect

Answer: C. Surgical resection and reconstruction

Explanation: For advanced osteoradionecrosis, the most definitive treatment is surgical resection of the necrotic bone followed by reconstruction. This approach removes the affected tissue and restores both form and function.

Question 50: Which genetic disorder is most commonly associated with multiple odontogenic keratocysts?

Solution:

1. Consider genetic disorders with oral manifestations

2. Review those associated with multiple cysts

3. Identify which specifically involves odontogenic keratocysts

Answer: D. Gorlin syndrome (Nevoid Basal Cell Carcinoma Syndrome)

Explanation: Gorlin syndrome, also known as Nevoid Basal Cell Carcinoma Syndrome, is characterized by multiple odontogenic keratocysts, along with other features like basal cell carcinomas and skeletal abnormalities.
