# Supplementary material for: How valuable are the questions and answers generated by large language models in oral and maxillofacial surgery?
Source: PLoS One. 2025 May 28;20(5):e0322529. doi: 10.1371/journal.pone.0322529 (PMC12119001; doi:10.1371/journal.pone.0322529)
Supplement: S1 File — (ZIP) [file pone.0322529.s001.zip › claude 3/CO - gpt4o.docx]

Question 1: Which nerve is most commonly injured during a mandibular third molar extraction?

Solution:

1. Consider the anatomy of the mandible and surrounding nerves

2. Identify nerves in close proximity to the third molar region

3. Evaluate the risk of injury for each nerve during extraction

Answer: C. Inferior alveolar nerve

Explanation: The inferior alveolar nerve runs through the mandibular canal, which is often in close proximity to the roots of the third molar. During extraction, this nerve is at the highest risk of injury due to its location.

Question 2: What is the most common cause of maxillofacial trauma?

Solution:

1. Consider various causes of facial injuries

2. Evaluate the frequency and severity of different trauma mechanisms

3. Assess societal factors that might contribute to certain types of injuries

Answer: C. Motor vehicle accidents

Explanation: Motor vehicle accidents often involve high-impact forces and are a frequent cause of severe facial injuries. The prevalence of motor vehicles and the potential for high-speed collisions make this the leading cause of maxillofacial trauma.

Question 3: Which muscle is primarily responsible for elevating the mandible?

Solution:

1. Review the muscles of mastication

2. Consider the primary function of each muscle

3. Identify which muscle has the most significant role in jaw closure

Answer: B. Masseter

Explanation: The masseter is a powerful muscle that attaches to the mandible and is primarily responsible for elevating the jaw. Its large size and favorable leverage make it the primary muscle for this action.

Question 4: What is the primary reason for performing a Le Fort I osteotomy?

Solution:

1. Consider the anatomical location of the Le Fort I osteotomy

2. Review common maxillofacial deformities

3. Evaluate which conditions can be corrected by mobilizing the maxilla

Answer: B. To address maxillary hypoplasia

Explanation: Le Fort I osteotomy involves separating the maxilla from the rest of the facial skeleton, allowing for its repositioning. This procedure is primarily used to correct maxillary hypoplasia by advancing the maxilla.

Question 5: Why is antibiotic prophylaxis recommended before dental procedures in patients with a history of infective endocarditis?

Solution:

1. Consider the risk of bacteremia during dental procedures

2. Review the pathophysiology of infective endocarditis

3. Evaluate the potential consequences of bacterial seeding in at-risk patients

Answer: C. To prevent bacterial endocarditis

Explanation: Dental procedures can cause transient bacteremia. In patients with a history of infective endocarditis, there's a risk of bacteria seeding cardiac tissues. Antibiotic prophylaxis aims to prevent this bacterial seeding and reduce the risk of recurrent endocarditis.

Question 6: A 45-year-old patient presents with limited mouth opening and pain near the ear. Which condition is most likely?

Solution:

1. Consider the anatomy of the temporomandibular joint (TMJ)

2. Review conditions that affect jaw movement and cause periauricular pain

3. Evaluate the prevalence of these conditions in middle-aged adults

Answer: A. Temporomandibular joint disorder

Explanation: Limited mouth opening and pain near the ear are classic symptoms of temporomandibular joint disorder (TMD). This condition is common in middle-aged adults and often involves dysfunction of the TMJ and surrounding muscles.

Question 7: A 30-year-old male presents with a swelling in the floor of the mouth that increases in size during meals. What is the likely diagnosis?

Solution:

1. Consider the anatomy of the floor of the mouth

2. Review conditions that cause swelling in this area

3. Evaluate which conditions are affected by salivary flow

Answer: A. Sialolithiasis

Explanation: Sialolithiasis (salivary stones) typically causes swelling that increases during meals due to obstruction of salivary flow. The submandibular gland, which opens into the floor of the mouth, is the most common site for stone formation.

Question 8: An X-ray shows a radiolucent lesion at the apex of a non-vital tooth. What is the most likely diagnosis?

Solution:

1. Consider the relationship between non-vital teeth and periapical lesions

2. Review the radiographic appearance of common periapical pathologies

3. Evaluate the most frequent outcome of chronic pulpal infection

Answer: A. Periapical abscess

Explanation: A radiolucent lesion at the apex of a non-vital tooth is typically a periapical abscess. This results from chronic infection of the pulp, leading to inflammation and bone resorption at the root apex.

Question 9: A CT scan reveals a "ground glass" appearance of the maxillary sinus. What condition does this suggest?

Solution:

1. Consider conditions that affect bone density

2. Review the radiographic appearance of various bone disorders

3. Evaluate which condition specifically produces a "ground glass" appearance

Answer: C. Fibrous dysplasia

Explanation: The "ground glass" appearance on CT is characteristic of fibrous dysplasia. This benign condition replaces normal bone with fibrous tissue, creating a distinctive radiographic appearance resembling ground glass.

Question 10: A patient presents with unilateral jaw pain, limited mouth opening, and a recent history of trauma. What imaging is most appropriate?

Solution:

1. Consider the potential injuries from facial trauma

2. Review the capabilities of different imaging modalities

3. Evaluate which imaging technique best visualizes both soft tissue and bone

Answer: C. CT scan

Explanation: CT scan is most appropriate in this case as it provides detailed images of both bony structures and soft tissues. It can reveal fractures, dislocations, and soft tissue injuries, making it ideal for evaluating facial trauma.

Question 11: Which artery is the primary blood supply to the mandible?

Solution:

1. Review the arterial anatomy of the lower face

2. Consider the course of arteries in relation to the mandible

3. Identify which artery runs within the mandibular canal

Answer: B. Inferior alveolar artery

Explanation: The inferior alveolar artery, a branch of the maxillary artery, is the primary blood supply to the mandible. It runs through the mandibular canal, supplying the mandibular teeth and surrounding bone.

Question 12: What is the primary treatment for an ameloblastoma?

Solution:

1. Consider the nature of ameloblastomas (locally aggressive, high recurrence rate)

2. Review treatment options for benign but aggressive tumors

3. Evaluate which approach best prevents recurrence

Answer: D. Wide local excision

Explanation: Wide local excision is the primary treatment for ameloblastomas due to their locally aggressive nature and high recurrence rate. This approach ensures complete removal of the tumor and reduces the risk of recurrence.

Question 13: A panoramic radiograph shows a well-defined, unilocular radiolucency associated with an unerupted third molar. What is the most likely diagnosis?

Solution:

1. Consider common lesions associated with unerupted teeth

2. Review the radiographic appearance of odontogenic cysts

3. Evaluate which lesion typically presents as a unilocular radiolucency around the crown of an unerupted tooth

Answer: B. Dentigerous cyst

Explanation: A well-defined, unilocular radiolucency associated with the crown of an unerupted tooth is characteristic of a dentigerous cyst. These cysts develop from the dental follicle of unerupted teeth, most commonly third molars.

Question 14: What is the most common benign salivary gland tumor?

Solution:

1. Review types of salivary gland tumors

2. Consider the frequency of occurrence for each type

3. Identify which benign tumor is most prevalent

Answer: C. Pleomorphic adenoma

Explanation: Pleomorphic adenoma is the most common benign salivary gland tumor. It typically occurs in the parotid gland and is characterized by slow growth and a mixed histological appearance.

Question 15: Which muscle assists in the retraction of the mandible?

Solution:

1. Review the muscles of mastication

2. Consider the direction of muscle fibers and their attachments

3. Identify which muscle has fibers oriented to pull the mandible posteriorly

Answer: D. Temporalis

Explanation: The temporalis muscle, particularly its posterior fibers, assists in retracting the mandible. While its primary function is elevation, the orientation of its posterior fibers allows it to pull the mandible backwards.

Question 16: In what situation is an orthognathic surgery typically indicated?

Solution:

1. Consider conditions that affect jaw alignment and facial proportions

2. Review indications for surgical intervention in maxillofacial deformities

3. Evaluate which condition might require significant jaw repositioning for treatment

Answer: B. Severe obstructive sleep apnea

Explanation: Orthognathic surgery is often indicated in cases of severe obstructive sleep apnea where the jaw position contributes to airway obstruction. Repositioning the jaws can help increase the airway space and improve breathing during sleep.

Question 17: A CT scan shows a "sunburst" pattern in the jaw. What condition does this suggest?

Solution:

1. Consider aggressive bone lesions that affect the jaws

2. Review radiographic patterns associated with malignant bone tumors

3. Identify which condition typically produces a "sunburst" appearance

Answer: B. Osteosarcoma

Explanation: A "sunburst" pattern on CT is highly suggestive of osteosarcoma. This aggressive malignant bone tumor produces rapid, irregular bone formation, resulting in the characteristic sunburst or spiculated appearance on imaging.

Question 18: A panoramic radiograph shows bilateral radiopaque areas in the mandibular angle region. What is the likely diagnosis?

Solution:

1. Consider common bony lesions or variations in the mandible

2. Review conditions that present as bilateral, radiopaque findings

3. Evaluate which condition typically occurs in the mandibular angle region

Answer: B. Tori mandibularis

Explanation: Bilateral radiopaque areas in the mandibular angle region are characteristic of tori mandibularis. These are benign bony outgrowths that commonly occur bilaterally in the lingual aspect of the mandible.

Question 19: A 60-year-old male presents with a painful, non-healing ulcer on the lateral border of the tongue. What is the most likely diagnosis?

Solution:

1. Consider risk factors (age, gender) for oral malignancies

2. Review common sites for oral cancer

3. Evaluate the clinical presentation of oral squamous cell carcinoma

Answer: A. Squamous cell carcinoma

Explanation: In an older male, a painful, non-healing ulcer on the lateral border of the tongue is highly suspicious for squamous cell carcinoma. This is a common site for oral cancer, and the non-healing nature of the lesion is concerning for malignancy.

Question 20: What is the most common complication following a mandibular fracture?

Solution:

1. Consider the anatomy and function of the mandible

2. Review potential complications of mandibular fractures

3. Evaluate which complication most directly affects jaw function

Answer: B. Malocclusion

Explanation: Malocclusion is the most common complication following mandibular fractures. Displacement of the fractured segments can alter the alignment of the teeth, leading to changes in the bite and overall occlusion.

Question 21: Which imaging modality is best for evaluating soft tissue structures of the temporomandibular joint?

Solution:

1. Consider the anatomy of the TMJ, including the articular disc

2. Review capabilities of different imaging modalities for soft tissue visualization

3. Evaluate which modality provides the best detail of soft tissues without radiation

Answer: B. MRI

Explanation: MRI is the best imaging modality for evaluating soft tissue structures of the TMJ. It provides excellent visualization of the articular disc, ligaments, and muscles without using ionizing radiation.

Question 22: A patient presents with trismus, fever, and swelling of the submandibular space. What is the most likely diagnosis?

Solution:

1. Consider infections that affect the submandibular space

2. Review symptoms of severe oral and facial infections

3. Evaluate which condition can cause rapid, severe swelling and trismus

Answer: A. Ludwig's angina

Explanation: Ludwig's angina is a severe infection of the submandibular space that typically presents with trismus, fever, and rapid swelling of the floor of the mouth and neck. It's a potentially life-threatening condition requiring immediate treatment.

Question 23: What is the gold standard treatment for a non-displaced mandibular condyle fracture?

Solution:

1. Consider the healing potential of condylar fractures

2. Review treatment options for mandibular fractures

3. Evaluate the risks and benefits of surgical vs. non-surgical management

Answer: C. Conservative management with a soft diet

Explanation: For non-displaced mandibular condyle fractures, conservative management with a soft diet is typically the gold standard. This allows for healing while maintaining function and avoiding the risks associated with surgery.

Question 24: A patient complains of persistent numbness in the lower lip after wisdom tooth extraction. Which nerve is likely affected?

Solution:

1. Review the anatomy of the nerves in the lower jaw

2. Consider which nerves are at risk during wisdom tooth extraction

3. Identify which nerve supplies sensation to the lower lip

Answer: B. Inferior alveolar nerve

Explanation: The inferior alveolar nerve is likely affected. It runs through the mandibular canal near the roots of the lower wisdom teeth and provides sensation to the lower lip. Injury during extraction can lead to numbness in this area.

Question 25: A panoramic radiograph reveals a mixed radiolucent-radiopaque lesion in the posterior mandible. What is the likely diagnosis?

Solution:

1. Consider lesions that can present with mixed radiolucent-radiopaque appearance

2. Review common locations for these lesions

3. Evaluate which condition typically occurs in the posterior mandible

Answer: E. Cemento-osseous dysplasia

Explanation: Cemento-osseous dysplasia often presents as a mixed radiolucent-radiopaque lesion in the posterior mandible. It goes through stages from radiolucent to mixed to radiopaque, making the mixed appearance a common finding.

Question 26: Which condition is characterized by the triad of craniofacial dysostosis, syndactyly, and mental retardation?

Solution:

1. Review genetic syndromes affecting craniofacial development

2. Consider conditions that affect both the skull and limbs

3. Identify which syndrome specifically includes these three features

Answer: B. Apert syndrome

Explanation: Apert syndrome is characterized by this triad of craniofacial dysostosis (premature fusion of skull sutures), syndactyly (fusion of fingers and/or toes), and varying degrees of intellectual disability.

Question 27: What is the typical presentation of osteoradionecrosis of the jaw?

Solution:

1. Consider the effects of radiation on bone

2. Review the clinical and radiographic signs of bone necrosis

3. Evaluate the progressive nature of the condition

Answer: E. All of the above (Painful, non-healing ulcer; Radiopaque mass; Soft tissue swelling; Fistula formation)

Explanation: Osteoradionecrosis can present with all these features. It typically begins with pain and exposed bone, progressing to ulceration, swelling, and fistula formation. Radiographically, it can appear as a radiopaque mass due to bone sclerosis.

Question 28: Which medication is commonly associated with medication-related osteonecrosis of the jaw (MRONJ)?

Solution:

1. Consider medications that affect bone metabolism

2. Review drugs used in treatment of osteoporosis and bone metastases

3. Identify which class of drugs has been most commonly linked to MRONJ

Answer: C. Bisphosphonates

Explanation: Bisphosphonates are most commonly associated with MRONJ. These drugs, used to treat osteoporosis and bone metastases, can interfere with bone remodeling and healing, leading to osteonecrosis.

Question 29: Which cranial nerve is primarily responsible for sensation in the face?

Solution:

1. Review the functions of cranial nerves

2. Consider which nerves have sensory components in the face

3. Identify the nerve with the most extensive sensory distribution in the face

Answer: B. Trigeminal nerve

Explanation: The trigeminal nerve (cranial nerve V) is primarily responsible for sensation in the face. It has three branches (ophthalmic, maxillary, and mandibular) that provide sensory innervation to most of the face.

Question 30: A 55-year-old patient presents with an asymptomatic swelling in the hard palate that has been gradually increasing in size. What is the most likely diagnosis?

Solution:

1. Consider common benign lesions of the hard palate

2. Review conditions that present as slow-growing, asymptomatic swellings

3. Evaluate which condition is most common in middle-aged adults

Answer: D. Torus palatinus

Explanation: Torus palatinus is the most likely diagnosis. It's a common, benign bony outgrowth on the hard palate that typically presents as a slow-growing, asymptomatic swelling in adults.

Question 31: Which condition is most likely to present with "onion skin" periosteal reaction on radiographs?

Solution:

1. Consider conditions that cause aggressive periosteal reactions

2. Review the radiographic appearance of bone tumors

3. Identify which condition specifically produces an "onion skin" pattern

Answer: B. Ewing's sarcoma

Explanation: Ewing's sarcoma typically presents with an "onion skin" periosteal reaction on radiographs. This appearance is due to layers of new bone formation in response to the rapidly growing tumor.

Question 32: A panoramic radiograph shows multiple radiolucent lesions in the mandible resembling soap bubbles. What is the likely diagnosis?

Solution:

1. Consider multilocular radiolucent lesions of the jaw

2. Review the radiographic appearance of odontogenic tumors

3. Identify which condition typically presents with a "soap bubble" appearance

Answer: A. Ameloblastoma

Explanation: Ameloblastoma often presents as a multilocular radiolucent lesion with a "soap bubble" or "honeycomb" appearance on radiographs. This is due to its slow-growing nature and expansion of cortical bone.

Question 33: Which condition is characterized by "cotton wool" appearance on radiographs?

Solution:

1. Consider conditions that affect bone density

2. Review radiographic patterns of metabolic bone diseases

3. Identify which condition produces a distinctive "cotton wool" appearance

Answer: C. Paget's disease

Explanation: Paget's disease of bone is characterized by a "cotton wool" appearance on radiographs. This is due to the alternating areas of bone resorption and formation, creating a mottled, fluffy appearance.

Question 34: A patient with a history of multiple dental extractions presents with a non-healing ulcer and bone exposure in the mandible. What is the likely diagnosis?

Solution:

1. Consider conditions that impair bone healing

2. Review risk factors for osteonecrosis of the jaw

3. Evaluate which condition is associated with dental extractions and medication use

Answer: B. MRONJ (Medication-Related Osteonecrosis of the Jaw)

Explanation: MRONJ is likely in a patient with a history of dental extractions presenting with non-healing ulcers and exposed bone. This condition is often associated with certain medications, particularly bisphosphonates.

Question 35: Which condition is associated with "ground glass" appearance on radiographs?

Solution:

1. Consider conditions that affect bone density and structure

2. Review radiographic patterns of benign bone lesions

3. Identify which condition produces a homogeneous, "ground glass" appearance

Answer: B. Fibrous dysplasia

Explanation: Fibrous dysplasia typically presents with a "ground glass" appearance on radiographs. This is due to the replacement of normal bone with fibrous tissue, creating a homogeneous, hazy appearance.

Question 36: A patient presents with a painless, slow-growing mass in the parotid gland. What is the most likely diagnosis?

Answer: A. Pleomorphic adenoma

Explanation: Pleomorphic adenoma is the most common benign tumor of the parotid gland, typically presenting as a painless, slow-growing mass.

Question 37: Which anatomical structure is at risk of injury during a submandibular gland excision?

Answer: D. Lingual nerve

Explanation: The lingual nerve runs close to the submandibular gland and is at risk during its excision.

Question 38: What is the first-line treatment for a patient presenting with a deep neck space infection?

Answer: C. Intravenous antibiotics

Explanation: Deep neck space infections require immediate treatment with intravenous antibiotics due to their potential for rapid spread and airway compromise.

Question 39: Which condition is characterized by the presence of "floating teeth" on radiographs?

Answer: A. Langerhans cell histiocytosis

Explanation: Langerhans cell histiocytosis can cause extensive alveolar bone destruction, giving the appearance of "floating teeth" on radiographs.

Question 40: A 40-year-old patient presents with pain and swelling in the lower jaw after a recent tooth extraction. What is the most likely diagnosis?

Answer: A. Dry socket (alveolar osteitis)

Explanation: Dry socket is a common complication following tooth extraction, characterized by pain and inflammation without signs of infection.

Question 41: What is the primary concern in a patient with bilateral mandibular fractures?

Answer: A. Airway obstruction

Explanation: Bilateral mandibular fractures can lead to posterior displacement of the fractured segments, potentially causing airway obstruction.

Question 42: A panoramic radiograph shows a multilocular radiolucent lesion in the maxilla with displacement of adjacent teeth. What is the likely diagnosis?

Answer: B. Odontogenic myxoma

Explanation: Odontogenic myxomas typically present as multilocular radiolucent lesions that can cause displacement of adjacent teeth.

Question 43: Which of the following is a potential complication of untreated maxillary sinusitis?

Answer: E. All of the above (Orbital cellulitis, Meningitis, Brain abscess, Osteomyelitis of the maxilla)

Explanation: Untreated maxillary sinusitis can lead to various serious complications due to the spread of infection to adjacent structures.

Question 44: What is the most common cause of mandibular prognathism?

Answer: A. Genetic factors

Explanation: Mandibular prognathism is most commonly caused by genetic factors influencing jaw growth and development.

Question 45: A patient presents with a painless, firm, and immobile mass in the anterior floor of the mouth. What is the most likely diagnosis?

Answer: B. Dermoid cyst

Explanation: Dermoid cysts commonly present as painless, firm masses in the midline of the anterior floor of the mouth.

Question 46: Which imaging modality is best for evaluating the extent of a salivary gland tumor?

Answer: B. MRI

Explanation: MRI provides excellent soft tissue contrast, making it ideal for evaluating the extent of salivary gland tumors.

Question 47: A 50-year-old male presents with difficulty swallowing, hoarseness, and a mass in the neck. What is the most likely diagnosis?

Answer: B. Laryngeal cancer

Explanation: These symptoms in an older male are highly suggestive of laryngeal cancer.

Question 48: A patient presents with a radiopaque lesion in the mandible that shows a "sunburst" pattern on imaging. What is the likely diagnosis?

Answer: A. Osteosarcoma

Explanation: A "sunburst" pattern is characteristic of osteosarcoma due to the rapid, irregular new bone formation.

Question 49: What is the most common site for mandibular fractures?

Answer: A. Condyle

Explanation: The condyle is the most common site for mandibular fractures due to its vulnerable position and anatomy.

Question 50: A patient presents with chronic sinusitis and a radiopaque mass in the maxillary sinus on CT scan. What is the most likely diagnosis?

Answer: D. Osteoma

Explanation: Osteomas are benign bone tumors that can occur in the paranasal sinuses and may present as radiopaque masses associated with chronic sinusitis.
