# Supplementary material for: How valuable are the questions and answers generated by large language models in oral and maxillofacial surgery?
Source: PLoS One. 2025 May 28;20(5):e0322529. doi: 10.1371/journal.pone.0322529 (PMC12119001; doi:10.1371/journal.pone.0322529)
Supplement: S1 File — (ZIP) [file pone.0322529.s001.zip › gpt4/gpt4-CO.docx]

1. **Which of the following is the most common site for oral cancer?**

- a) Buccal mucosa

- b) Floor of the mouth

- c) Hard palate

- d) Tongue

- e) Gingiva

**Answer: d) Tongue**

**Explanation:** Studies indicate that the tongue is the most common site for oral cancer, accounting for a significant portion of oral cancer cases.

2. **A 45-year-old male presents with a painless, firm, and fixed lymph node in the submandibular region. What is the most likely diagnosis?**

- a) Reactive lymphadenitis

- b) Sialadenitis

- c) Lymphoma

- d) Metastatic oral cancer

- e) Lipoma

**Answer: d) Metastatic oral cancer**

**Explanation:** A painless, firm, and fixed lymph node suggests a malignant condition, and given the location and patient profile, metastatic oral cancer is the most likely diagnosis.

3. **Which of the following is the most common type of odontogenic tumor?**

- a) Ameloblastoma

- b) Odontoma

- c) Odontogenic myxoma

- d) Adenomatoid odontogenic tumor

- e) Calcifying epithelial odontogenic tumor

**Answer: b) Odontoma**

**Explanation:** Odontomas are the most common type of odontogenic tumor, often being benign and presenting as complex or compound types.

4. **A patient presents with a unilateral, painful swelling in the preauricular region that is exacerbated by eating. What is the most likely diagnosis?**

- a) Mumps

- b) Sialolithiasis

- c) Sjögren's syndrome

- d) Parotid gland tumor

- e) Temporomandibular joint disorder

**Answer: b) Sialolithiasis**

**Explanation:** Painful swelling that worsens during eating is typical of sialolithiasis, caused by salivary gland stones obstructing the salivary flow.

5. **Which of the following is the most common site for maxillofacial fractures?**

- a) Nasal bones

- b) Zygoma

- c) Mandible

- d) Orbital floor

- e) Maxilla

**Answer: c) Mandible**

**Explanation:** The mandible is the most commonly fractured bone in the facial skeleton due to its prominence and exposure to trauma.

6. **A patient presents with a painless, slow-growing mass in the parotid gland. Which of the following is the most likely diagnosis?**

- a) Pleomorphic adenoma

- b) Warthin's tumor

- c) Mucoepidermoid carcinoma

- d) Adenoid cystic carcinoma

- e) Acinic cell carcinoma

**Answer: a) Pleomorphic adenoma**

**Explanation:** Pleomorphic adenoma is the most common benign tumor of the salivary glands, typically presenting as a painless, slow-growing mass.

7. **Which of the following nerves is most commonly injured during third molar extraction?**

- a) Facial nerve

- b) Glossopharyngeal nerve

- c) Hypoglossal nerve

- d) Inferior alveolar nerve

- e) Lingual nerve

**Answer: d) Inferior alveolar nerve**

**Explanation:** The inferior alveolar nerve is at significant risk during third molar (wisdom tooth) extractions due to its path in the mandibular canal.

8. **A patient presents with a unilateral, painful, erythematous swelling in the submandibular region. Which of the following is the most likely diagnosis?**

- a) Submandibular sialolithiasis

- b) Submandibular sialadenitis

- c) Submandibular gland tumor

- d) Cervical lymphadenitis

- e) Ranula

**Answer: b) Submandibular sialadenitis**

**Explanation:** Painful inflammation of the submandibular gland, commonly accompanied by erythema, points toward sialadenitis.

9. **Which of the following is the most common type of ameloblastoma?**

- a) Unicystic ameloblastoma

- b) Solid/multicystic ameloblastoma

- c) Peripheral ameloblastoma

- d) Desmoplastic ameloblastoma

- e) Malignant ameloblastoma

**Answer: b) Solid/multicystic ameloblastoma**

**Explanation:** Solid/multicystic ameloblastomas are the most common type, characterized by multiple cystic spaces.

10. **A patient presents with a painful, non-healing ulcer on the lateral border of the tongue that has been present for 6 weeks. Which of the following is the most appropriate next step in management?**

- a) Prescribe antibiotics

- b) Perform an incisional biopsy

- c) Refer for CT scan

- d) Prescribe topical corticosteroids

- e) Reassure and observe

**Answer: b) Perform an incisional biopsy**

**Explanation:** A non-healing ulcer lasting more than 2 weeks should be biopsied to rule out malignancy, especially in high-risk sites like the lateral tongue.

11. **Which of the following is the most common site for osteonecrosis of the jaw associated with bisphosphonate therapy?**

- a) Maxilla

- b) Mandible

- c) Zygoma

- d) Nasal bones

- e) Frontal bone

**Answer: b) Mandible**

**Explanation:** The mandible is more commonly affected by bisphosphonate-related osteonecrosis due to its dense bone and lower blood supply compared to the maxilla.

12. **A patient presents with a rapidly growing, painful swelling in the mandible. Radiographic examination reveals a multilocular radiolucency with irregular borders. Which of the following is the most likely diagnosis?**

- a) Ameloblastoma

- b) Odontogenic keratocyst

- c) Central giant cell granuloma

- d) Dentigerous cyst

- e) Osteosarcoma

**Answer: e) Osteosarcoma**

**Explanation:** A rapidly growing, painful jaw swelling with multilocular radiolucency and irregular borders is characteristic of osteosarcoma, a malignant bone tumor.

13. **Which of the following is the most common type of maxillofacial trauma in pediatric patients?**

- a) Mandibular fractures

- b) Zygomatic fractures

- c) Nasal fractures

- d) Orbital fractures

- e) Frontal bone fractures

**Answer: c) Nasal fractures**

**Explanation:** Due to the prominence and fragility of the nasal bones, nasal fractures are the most common facial injury in children.

14. **A patient presents with a painless, slow-growing mass in the submandibular gland. Fine needle aspiration biopsy reveals a mixture of epithelial and myoepithelial cells. Which of the following is the most likely diagnosis?**

- a) Pleomorphic adenoma

- b) Warthin's tumor

- c) Mucoepidermoid carcinoma

- d) Adenoid cystic carcinoma

- e) Acinic cell carcinoma

**Answer: a) Pleomorphic adenoma**

**Explanation:** Pleomorphic adenoma typically shows a mixture of epithelial and myoepithelial cells on fine needle aspiration and is the most common benign salivary gland tumor.

15. **Which of the following is the most common site for cervical lymph node metastasis in oral squamous cell carcinoma?**

- a) Level I (submandibular and submental nodes)

- b) Level II (upper jugular nodes)

- c) Level III (middle jugular nodes)

- d) Level IV (lower jugular nodes)

- e) Level V (posterior triangle nodes)

**Answer: b) Level II (upper jugular nodes)**

**Explanation:** Level II nodes are frequently the first site of metastasis for oral squamous cell carcinoma.

16. **A patient presents with a unilateral, diffuse swelling of the parotid gland associated with dry mouth and dry eyes. Which of the following is the most likely diagnosis?**

- a) Sjögren's syndrome

- b) Sialolithiasis

- c) Sialadenitis

- d) Parotid gland tumor

- e) Sarcoidosis

**Answer: a) Sjögren's syndrome**

**Explanation:** Sjögren's syndrome causes lymphocytic infiltration of the salivary and lacrimal glands, leading to swelling, dry mouth, and dry eyes.

17. **Which of the following is the most common type of odontogenic cyst?**

- a) Radicular cyst

- b) Dentigerous cyst

- c) Odontogenic keratocyst

- d) Lateral periodontal cyst

- e) Glandular odontogenic cyst

**Answer: a) Radicular cyst**

**Explanation:** Radicular cysts, arising from non-vital teeth, are the most common odontogenic cysts.

18. **A patient presents with a firm, painless mass in the floor of the mouth that causes elevation of the tongue. Which of the following is the most likely diagnosis?**

- a) Ranula

- b) Dermoid cyst

- c) Lipoma

- d) Pleomorphic adenoma

- e) Mucoepidermoid carcinoma

**Answer: b) Dermoid cyst**

**Explanation:** Dermoid cysts commonly present as firm, painless masses that can cause tongue elevation.

19. **Which of the following is the most common site for osteoradionecrosis of the jaws?**

- a) Maxilla

- b) Mandible

- c) Zygoma

- d) Nasal bones

- e) Frontal bone

**Answer: b) Mandible**

**Explanation:** The mandible is more frequently affected by osteoradionecrosis due to its relatively poor blood supply and dense cortical bone structure.

20. **A patient presents with a unilateral, fluctuant swelling in the buccal vestibule adjacent to a vital tooth. Radiographic examination reveals no abnormalities. Which of the following is the most likely diagnosis?**

- a) Radicular cyst

- b) Dentigerous cyst

- c) Nasolabial cyst

- d) Odontogenic keratocyst

- e) Lateral periodontal cyst

**Answer: c) Nasolabial cyst**

**Explanation:** Nasolabial cysts typically present as fluctuant swellings in the buccal vestibule without radiographic changes and are not associated with teeth.

21. **Which of the following is the most common site for a lingual thyroid?**

- a) Anterior tongue

- b) Posterior tongue

- c) Lateral tongue

- d) Floor of the mouth

- e) Soft palate

**Answer: b) Posterior tongue**

**Explanation:** Lingual thyroid tissue is most commonly found at the base of the tongue, in the region of the foramen cecum.

22. **A patient presents with a rapidly growing, painless mass in the parotid gland. Fine needle aspiration biopsy reveals a predominance of lymphoid cells. Which of the following is the most likely diagnosis?**

- a) Pleomorphic adenoma

- b) Warthin's tumor

- c) Mucoepidermoid carcinoma

- d) Adenoid cystic carcinoma

- e) Acinic cell carcinoma

**Answer: b) Warthin's tumor**

**Explanation:** Warthin's tumor (papillary cystadenoma lymphomatosum) typically presents as a painless mass in the parotid gland with a significant lymphoid component on histology.

23. **Which of the following is the most common type of maxillofacial fracture in elderly patients?**

- a) Mandibular fractures

- b) Zygomatic fractures

- c) Nasal fractures

- d) Orbital fractures

- e) Maxillary fractures

**Answer: a) Mandibular fractures**

**Explanation:** In elderly patients, mandibular fractures are common due to increased bone fragility and susceptibility to falls.

24. **A patient presents with a slow-growing, painless mass in the submandibular gland. Fine needle aspiration biopsy reveals a predominance of uniform, basaloid cells with a cribriform pattern. Which of the following is the most likely diagnosis?**

- a) Pleomorphic adenoma

- b) Warthin's tumor

- c) Mucoepidermoid carcinoma

- d) Adenoid cystic carcinoma

- e) Acinic cell carcinoma

**Answer: d) Adenoid cystic carcinoma**

**Explanation:** Adenoid cystic carcinoma presents with basaloid cells and a cribriform pattern, often in the submandibular gland.

25. **Which of the following is the most common site for a dentigerous cyst?**

- a) Maxillary anterior region

- b) Maxillary posterior region

- c) Mandibular anterior region

- d) Mandibular posterior region

- e) Mandibular ramus

**Answer: d) Mandibular posterior region**

**Explanation:** Dentigerous cysts commonly occur around impacted mandibular third molars in the posterior region.

I'll stop here for now to keep the response manageable. If you need further questions or all the questions addressed, please let me know!

Sure, here are the solutions for the remaining questions:

26. **A patient presents with a diffuse, bilateral swelling of the parotid glands associated with fever, malaise, and myalgia. Which of the following is the most likely diagnosis?**

- a) Sjögren's syndrome

- b) Sialolithiasis

- c) Sialadenitis

- d) Parotid gland tumor

- e) Mumps

**Answer: e) Mumps**

**Explanation:** Mumps is a viral infection characterized by bilateral parotid gland swelling, fever, malaise, and myalgia. It is caused by the mumps virus (a paramyxovirus).

27. **Which of the following is the most common type of odontogenic tumor in children?**

- a) Ameloblastoma

- b) Odontoma

- c) Odontogenic myxoma

- d) Adenomatoid odontogenic tumor

- e) Calcifying epithelial odontogenic tumor

**Answer: b) Odontoma**

**Explanation:** Odontomas are the most common type of odontogenic tumor in children, frequently presenting as mixed radiopaque lesions on radiographs.

28. **A patient presents with a painful, non-healing ulcer on the ventral surface of the tongue that has been present for 3 months. The patient has a history of heavy smoking and alcohol consumption. Which of the following is the most appropriate next step in management?**

- a) Prescribe antibiotics

- b) Perform an incisional biopsy

- c) Refer for CT scan

- d) Prescribe topical corticosteroids

- e) Reassure and observe

**Answer: b) Perform an incisional biopsy**

**Explanation:** A non-healing ulcer on the tongue, especially with a history of smoking and alcohol consumption, is highly suspicious for oral cancer. A biopsy is needed for a definitive diagnosis.

29. **Which of the following is the most common site for a ranula?**

- a) Buccal mucosa

- b) Floor of the mouth

- c) Hard palate

- d) Tongue

- e) Gingiva

**Answer: b) Floor of the mouth**

**Explanation:** Ranulas are mucoceles that arise in the floor of the mouth, typically resulting from the rupture of the sublingual gland ducts.

30. **A patient presents with a rapidly growing, painful mass in the mandible. Radiographic examination reveals a radiolucent lesion with irregular borders and a "sunburst" appearance. Which of the following is the most likely diagnosis?**

- a) Ameloblastoma

- b) Odontogenic keratocyst

- c) Central giant cell granuloma

- d) Dentigerous cyst

- e) Osteosarcoma

**Answer: e) Osteosarcoma**

**Explanation:** A rapidly growing, painful mass with a "sunburst" radiographic appearance and irregular borders is characteristic of osteosarcoma, a highly aggressive bone tumor.

31. **Which of the following is the most common type of maxillofacial trauma in adult patients?**

- a) Mandibular fractures

- b) Zygomatic fractures

- c) Nasal fractures

- d) Orbital fractures

- e) Frontal bone fractures

**Answer: a) Mandibular fractures**

**Explanation:** Mandibular fractures are common in adults due to the prominence of the jaw and exposure to trauma.

32. **A patient presents with a slow-growing, painless mass in the parotid gland. Fine needle aspiration biopsy reveals a predominance of mucous cells. Which of the following is the most likely diagnosis?**

- a) Pleomorphic adenoma

- b) Warthin's tumor

- c) Mucoepidermoid carcinoma

- d) Adenoid cystic carcinoma

- e) Acinic cell carcinoma

**Answer: c) Mucoepidermoid carcinoma**

**Explanation:** A predominance of mucous cells on fine needle aspiration suggests mucoepidermoid carcinoma, the most common malignant salivary gland tumor.

33. **Which of the following is the most common site for a nasopalatine duct cyst?**

- a) Maxillary anterior region

- b) Maxillary posterior region

- c) Mandibular anterior region

- d) Mandibular posterior region

- e) Soft palate

**Answer: a) Maxillary anterior region**

**Explanation:** Nasopalatine duct cysts typically occur in the maxillary anterior region, specifically within the incisive canal.

34. **A patient presents with a firm, painless mass in the tongue that has been slowly growing for 6 months. Which of the following is the most likely diagnosis?**

- a) Squamous cell carcinoma

- b) Fibroma

- c) Lipoma

- d) Hemangioma

- e) Lymphangioma

**Answer: b) Fibroma**

**Explanation:** A firm, painless mass in the tongue growing slowly over months suggests a fibroma, a benign fibrous tissue tumor.

35. **Which of the following is the most common type of odontogenic tumor in adults?**

- a) Ameloblastoma

- b) Odontoma

- c) Odontogenic myxoma

- d) Adenomatoid odontogenic tumor

- e) Calcifying epithelial odontogenic tumor

**Answer: a) Ameloblastoma**

**Explanation:** Ameloblastomas are common odontogenic tumors in adults, known for being locally aggressive and often requiring surgical removal.

36. **A patient presents with a unilateral, painful swelling in the submandibular region that is exacerbated by eating. Radiographic examination reveals a radiopaque mass within the submandibular duct. Which of the following is the most likely diagnosis?**

- a) Submandibular sialolithiasis

- b) Submandibular sialadenitis

- c) Submandibular gland tumor

- d) Cervical lymphadenitis

- e) Ranula

**Answer: a) Submandibular sialolithiasis**

**Explanation:** A radiopaque mass within the submandibular duct, along with painful swelling during eating, is indicative of submandibular sialolithiasis (salivary gland stones).

37. **Which of the following is the most common site for a mucoepidermoid carcinoma?**

- a) Parotid gland

- b) Submandibular gland

- c) Sublingual gland

- d) Minor salivary glands of the palate

- e) Minor salivary glands of the buccal mucosa

**Answer: a) Parotid gland**

**Explanation:** Mucoepidermoid carcinoma frequently occurs in the parotid gland, making it the most common site for this type of tumor.

38. **A patient presents with a painful, non-healing ulcer on the lateral border of the tongue that has been present for 4 weeks. The patient has a history of chewing tobacco. Which of the following is the most appropriate next step in management?**

- a) Prescribe antibiotics

- b) Perform an incisional biopsy

- c) Refer for CT scan

- d) Prescribe topical corticosteroids

- e) Advise the patient to quit using tobacco products

**Answer: b) Perform an incisional biopsy**

**Explanation:** A non-healing ulcer in a tobacco user should be biopsied to rule out squamous cell carcinoma, given the high risk associated with tobacco use.

39. **Which of the following is the most common site for a pleomorphic adenoma?**

- a) Parotid gland

- b) Submandibular gland

- c) Sublingual gland

- d) Minor salivary glands of the palate

- e) Minor salivary glands of the lip

**Answer: a) Parotid gland**

**Explanation:** Pleomorphic adenomas most frequently occur in the parotid gland and are the most common benign salivary gland tumors.

40. **A patient presents with a rapidly growing, painless mass in the posterior mandible. Radiographic examination reveals a multilocular radiolucency with scalloped borders. Which of the following is the most likely diagnosis?**

- a) Ameloblastoma

- b) Odontogenic keratocyst

- c) Central giant cell granuloma

- d) Dentigerous cyst

- e) Odontogenic myxoma

**Answer: a) Ameloblastoma**

**Explanation:** Ameloblastomas typically present as multilocular radiolucencies with scalloped borders, particularly in the posterior mandible.

41. **Which of the following is the most common type of maxillofacial fracture resulting from motor vehicle accidents?**

- a) Mandibular fractures

- b) Zygomatic fractures

- c) Nasal fractures

- d) Orbital fractures

- e) Le Fort fractures

**Answer: e) Le Fort fractures**

**Explanation:** Le Fort fractures, particularly Le Fort I, II, and III, commonly result from the significant facial trauma seen in motor vehicle accidents.

42. **A patient presents with a slow-growing, painless mass in the parotid gland. Fine needle aspiration biopsy reveals a predominance of oncocytic cells. Which of the following is the most likely diagnosis?**

- a) Pleomorphic adenoma

- b) Warthin's tumor

- c) Mucoepidermoid carcinoma

- d) Adenoid cystic carcinoma

- e) Acinic cell carcinoma

**Answer: b) Warthin's tumor**

**Explanation:** Warthin's tumor is characterized by oncocytic cells and is a common benign tumor in the parotid gland.

43. **Which of the following is the most common site for a central giant cell granuloma?**

- a) Maxillary anterior region

- b) Maxillary posterior region

- c) Mandibular anterior region

- d) Mandibular posterior region

- e) Mandibular condyle

**Answer: d) Mandibular posterior region**

**Explanation:** Central giant cell granulomas are commonly found in the posterior region of the mandible.

44. **A patient presents with a diffuse, bilateral enlargement of the parotid glands associated with xerostomia and rheumatoid arthritis. Which of the following is the most likely diagnosis?**

- a) Sjögren's syndrome

- b) Sialolithiasis

- c) Sialadenitis

- d) Parotid gland tumors

- e) Sarcoidosis

**Answer: a) Sjögren's syndrome**

**Explanation:** Sjögren's syndrome typically presents with diffuse parotid gland enlargement and is often associated with systemic autoimmune diseases like rheumatoid arthritis.

45. **Which of the following is the most common type of odontogenic cyst associated with an impacted tooth?**

- a) Radicular cyst

- b) Dentigerous cyst

- c) Odontogenic keratocyst

- d) Lateral periodontal cyst

- e) Glandular odontogenic cyst

**Answer: b) Dentigerous cyst**

**Explanation:** Dentigerous cysts are often associated with impacted teeth, particularly third molars or canines.

46. **A patient presents with a painful, non-healing ulcer on the floor of the mouth that has been present for 2 months. The patient has a history of smoking and drinking alcohol. Which of the following is the most appropriate next step in management?**

- a) Prescribe antibiotics

- b) Perform an incisional biopsy

- c) Refer for CT scan

- d) Prescribe topical corticosteroids

- e) Advise the patient to quit smoking and drinking alcohol

**Answer: b) Perform an incisional biopsy**

**Explanation:** A non-healing ulcer in a high-risk location and patient demographic (smoking and alcohol use) warrants an incisional biopsy to rule out malignancy.

47. **Which of the following is the most common site for a mucocele?**

- a) Buccal mucosa

- b) Floor of the mouth

- c) Hard palate

- d) Tongue

- e) Lower lip

**Answer: e) Lower lip**

**Explanation:** Mucoceles most commonly occur on the lower lip due to minor salivary gland duct damage or blockage.

48. **A patient presents with a slow-growing, painless mass in the submandibular gland. Fine needle aspiration biopsy reveals a predominance of clear cells. Which of the following is the most likely diagnosis?**

- a) Pleomorphic adenoma

- b) Warthin's tumor

- c) Mucoepidermoid carcinoma

- d) Adenoid cystic carcinoma

- e) Acinic cell carcinoma

**Answer: e) Acinic cell carcinoma**

**Explanation:** Acinic cell carcinoma typically shows clear cells on biopsy and is a low-grade malignant tumor of the salivary glands.

49. **Which of the following is the most common site for a traumatic bone cyst?**

- a) Maxillary anterior region

- b) Maxillary posterior region

- c) Mandibular anterior region

- d) Mandibular posterior region

- e) Mandibular condyle

**Answer: d) Mandibular posterior region**

**Explanation:** Traumatic bone cysts are most frequently found in the mandibular posterior region.

50. **A patient presents with a rapidly growing, painful mass in the maxillary sinus. Radiographic examination reveals a radiopaque lesion with a "ground glass" appearance. Which of the following is the most likely diagnosis?**

- a) Osteoma

- b) Fibrous dysplasia

- c) Ossifying fibroma

- d) Osteosarcoma

- e) Chondrosarcoma

**Answer: b) Fibrous dysplasia**

**Explanation:** A rapidly growing, painful mass in the maxillary sinus with a "ground glass" radiographic appearance is indicative of fibrous dysplasia, a benign fibro-osseous lesion.
