# Supplementary material for: How valuable are the questions and answers generated by large language models in oral and maxillofacial surgery?
Source: PLoS One. 2025 May 28;20(5):e0322529. doi: 10.1371/journal.pone.0322529 (PMC12119001; doi:10.1371/journal.pone.0322529)
Supplement: S1 File — (ZIP) [file pone.0322529.s001.zip › gpt4/gpt4-gpt4.docx]

### Question 1

1. **Which nerve is most commonly injured during a mandibular third molar extraction?**

- A. Lingual nerve

- B. Buccal nerve

- C. Inferior alveolar nerve

- D. Mental nerve

- E. Facial nerve

- **Correct Answer**: A. Lingual nerve

- **Explanation**: Although the inferior alveolar nerve is close to the mandibular third molars, the lingual nerve is often in the direct path of surgical instruments and is more frequently injured during third molar extractions.

### Question 2

2. **A 45-year-old male presents with a fractured zygomatic arch. Which imaging modality is most suitable to evaluate the fracture?**

- A. Panoramic radiograph

- B. Cephalometric radiograph

- C. MRI

- D. CT scan

- E. Ultrasound

- **Correct Answer**: D. CT scan

- **Explanation**: CT scans provide detailed and accurate images of bony structures, making them ideal for evaluating fractures like those in the zygomatic arch.

### Question 3

3. **What is the most common cause of osteonecrosis of the jaw?**

- A. Diabetes mellitus

- B. Bisphosphonate therapy

- C. Steroid use

- D. Chemotherapy

- E. Trauma

- **Correct Answer**: B. Bisphosphonate therapy

- **Explanation**: Bisphosphonates are strongly associated with osteonecrosis of the jaw, especially when used long-term for conditions like osteoporosis or metastatic cancer.

### Question 4

4. **During TMJ surgical procedures, which structure is most at risk of injury?**

- A. Maxillary artery

- B. Facial nerve

- C. Lingual nerve

- D. Mandibular branch of the trigeminal nerve

- E. Inferior alveolar nerve

- **Correct Answer**: B. Facial nerve

- **Explanation**: The facial nerve is at significant risk during TMJ surgery due to its close anatomical location.

### Question 5

5. **What is the first-line surgical intervention for severe obstructive sleep apnea unresponsive to CPAP therapy?**

- A. Adenoidectomy

- B. Tonsillectomy

- C. Uvulopalatopharyngoplasty (UPPP)

- D. Genioglossus advancement

- E. Tracheostomy

- **Correct Answer**: C. Uvulopalatopharyngoplasty (UPPP)

- **Explanation**: UPPP is often performed to remove redundant tissue from the throat to improve airway patency.

### Question 6

6. **What is the typical imaging feature of a dentigerous cyst on a panoramic radiograph?**

- A. Radiopaque mass adjacent to the tooth

- B. Radiolucent area surrounding an unerupted tooth

- C. Mixed radiopaque and radiolucent pattern near the apices

- D. Radiolucent area with a sclerotic border independent of teeth

- E. Ground-glass appearance

- **Correct Answer**: B. Radiolucent area surrounding an unerupted tooth

- **Explanation**: A dentigerous cyst is defined by a radiolucent area around the crown of an unerupted tooth.

### Question 7

7. **In the case of a mandibular angle fracture, which of the following is the most crucial factor in deciding the treatment plan?**

- A. Patient's age

- B. Degree of displacement

- C. Presence of teeth in the fracture line

- D. Patient's medical history

- E. Patient's preference

- **Correct Answer**: B. Degree of displacement

- **Explanation**: The degree of displacement will guide whether surgical or conservative management is required.

### Question 8

8. **A 25-year-old female presents with a swelling at the angle of the mandible. On imaging, a well-circumscribed, radiolucent lesion with a “soap-bubble” appearance is identified. What is the most likely diagnosis?**

- A. Ameloblastoma

- B. Odontogenic keratocyst

- C. Dentigerous cyst

- D. Fibrous dysplasia

- E. Osteoma

- **Correct Answer**: A. Ameloblastoma

- **Explanation**: Ameloblastomas frequently exhibit a "soap-bubble" or multilocular radiographic appearance.

### Question 9

9. **What surgical technique is typically used to correct mandibular prognathism?**

- A. Le Fort I osteotomy

- B. Bilateral sagittal split osteotomy (BSSO)

- C. Genial osteotomy

- D. Genioplasty

- E. Condylectomy

- **Correct Answer**: B. Bilateral sagittal split osteotomy (BSSO)

- **Explanation**: BSSO is standard for repositioning the mandibular structure to correct prognathism.

### Question 10

10. **In the setting of facial trauma, which Le Fort fracture type involves separation of the midface from the cranial base?**

- A. Le Fort I

- B. Le Fort II

- C. Le Fort III

- D. Le Fort IV

- E. None of the above

- **Correct Answer**: C. Le Fort III

- **Explanation**: Le Fort III fractures result in a complete separation of the facial skeleton from the cranial base, often termed craniofacial disjunction.

### Question 11

11. **A 60-year-old patient presents with an oral squamous cell carcinoma involving the floor of the mouth. Which of the following is the most appropriate initial imaging modality for staging the tumor?**

- A. Panoramic radiograph

- B. Chest X-ray

- C. Ultrasound

- D. Contrast-enhanced CT scan

- E. MRI

- **Correct Answer**: D. Contrast-enhanced CT scan

- **Explanation**: Contrast-enhanced CT scans are essential for staging as they provide comprehensive details regarding tumor size, local invasion, and nodal involvement.

### Question 12

12. **Which condition is characterized by bilateral enlargement of the parotid glands and is often associated with chronic alcohol abuse?**

- A. Sjögren’s syndrome

- B. Sialolithiasis

- C. Sialadenitis

- D. Mikulicz disease

- E. Sialadenosis

- **Correct Answer**: E. Sialadenosis

- **Explanation**: Sialadenosis, usually non-inflammatory, leads to parotid gland enlargement and is associated with chronic alcohol use, diabetes, and malnutrition.

### Question 13

13. **What is the preferred surgical treatment for a benign tumor located at the angle of the mandible?**

- A. Enucleation

- B. Marginal resection

- C. Segmental resection

- D. Curettage

- E. Intralesional corticosteroid injection

- **Correct Answer**: B. Marginal resection

- **Explanation**: This approach involves removing the tumor with a slight margin of surrounding healthy tissue, ensuring complete excision while preserving bone continuity.

### Question 14

14. **A patient presents with a suspected mandibular fracture. Which physical examination finding is most indicative of this diagnosis?**

- A. Trismus

- B. Anterior open bite

- C. Deviation of the mandible on opening

- D. Submandibular ecchymosis

- E. Pain on lateral palpation of the TMJ

- **Correct Answer**: B. Anterior open bite

- **Explanation**: An anterior open bite often results from bilateral condylar fractures, disturbing the occlusion pattern.

### Question 15

15. **Which type of flap is commonly used for large defects in oral cancer reconstruction that require both soft tissue and bony reconstruction?**

- A. Buccal fat pad flap

- B. Radial forearm free flap

- C. Pectoralis major flap

- D. Fibula free flap

- E. Temporalis myofascial flap

- **Correct Answer**: D. Fibula free flap

- **Explanation**: The fibula free flap combines structural bone and soft tissue to repair extensive defects following oral cancer resection effectively.

### Question 16

16. **Which of these statements about odontogenic keratocyst (OKC) is true?**

- A. It is most commonly found in the maxilla.

- B. It has a high recurrence rate after treatment.

- C. It is a malignant cyst.

- D. It is always associated with impacted teeth.

- E. It is typically associated with cleidocranial dysplasia.

- **Correct Answer**: B. It has a high recurrence rate after treatment.

- **Explanation**: OKCs are notorious for their high recurrence rate due to their aggressive behavior and infiltrative growth.

### Question 17

17. **In orthognathic surgery, which of the following is considered when planning for a mandibular setback?**

- A. Overbite reduction

- B. Increase in mandibular angle

- C. Advancement of the maxilla

- D. Widening of the mandibular arch

- E. Improvement in interdisciplinary occlusion

- **Correct Answer**: B. Increase in mandibular angle

- **Explanation**: Mandibular setback involves repositioning the mandible to correct skeletal alignment, altering the mandibular angle and teeth positioning concerning the maxilla.

### Question 18

18. **Which characteristic feature is often seen in panoramic radiographs of patients with amelogenesis imperfecta?**

- A. Radiolucent lesions at the tooth apices

- B. Generalized thinning of enamel

- C. Mixed radiolucent-radiopaque lesions

- D. Ground-glass appearance of bone

- E. “Cotton wool” appearance

- **Correct Answer**: B. Generalized thinning of enamel

- **Explanation**: Amelogenesis imperfecta usually exhibits generalized thinning or absence of enamel, resulting in similar density levels between the enamel and dentin.

### Question 19

19. **A 35-year-old male is involved in a road traffic accident and presents with a midface fracture. The clinical and radiological findings suggest a zygomaticomaxillary complex fracture. Which clinical sign is most likely to be present?**

- A. Enophthalmos

- B. Subcutaneous emphysema

- C. Maxillary mucosal tear

- D. Inferior orbital rim step deformity

- E. Nasal deviation

- **Correct Answer**: D. Inferior orbital rim step deformity

- **Explanation**: A zygomaticomaxillary complex fracture often presents with an observable step-off deformity at the inferior orbital rim due to zygoma displacement.

### Question 20

20. **What is the main advantage of using intraoperative navigation systems in maxillofacial surgery?**

- A. Reduced surgical cost

- B. Shortened surgical time

- C. Enhanced preoperative planning

- D. Increased precision in anatomical localization

- E. Improved aesthetic outcomes

- **Correct Answer**: D. Increased precision in anatomical localization

- **Explanation**: Intraoperative navigation enhances surgical accuracy by providing real-time anatomical localization, significantly improving surgical precision and outcomes.

### Question 21

21. **Which muscle is most commonly involved in trismus following a mandibular third molar extraction?**

- A. Masseter

- B. Temporalis

- C. Medial pterygoid

- D. Lateral pterygoid

- E. Buccinator

- **Correct Answer**: C. Medial pterygoid

- **Explanation**: The medial pterygoid muscle is at higher risk due to its anatomical proximity to the mandibular third molar, often resulting in postoperative trismus.

### Question 22

22. **Which of the following is a common sign of an orbital floor fracture (blowout fracture)?**

- A. Hypoglobus

- B. Hyperglobus

- C. Enophthalmos

- D. Proptosis

- E. Diplopia with up-gaze

- **Correct Answer**: C. Enophthalmos

- **Explanation**: Orbital floor fractures typically lead to enophthalmos as the orbital contents lose support, causing the eyeball to sink into the orbital cavity.

### Question 23

23. **Which antibiotic is recommended as first-line therapy for odontogenic infections that are anaerobic in nature?**

- A. Penicillin

- B. Erythromycin

- C. Cephalexin

- D. Metronidazole

- E. Tetracycline

- **Correct Answer**: D. Metronidazole

- **Explanation**: Metronidazole targets anaerobic pathogens effectively, making it an optimal choice for odontogenic infections involving anaerobic bacteria.

### Question 24

24. **A panoramic radiograph of a patient reveals a radiopaque lesion with a radiolucent halo in the posterior mandible. What is the most likely diagnosis?**

- A. Ameloblastoma

- B. Cemento-osseous dysplasia

- C. Odontoma

- D. Osteosarcoma

- E. Dentigerous cyst

- **Correct Answer**: C. Odontoma

- **Explanation**: An odontoma frequently presents as a radiopaque lesion with a surrounding radiolucent halo, indicative of its composite nature of enamel and dentin.

### Question 25

25. **Which condition is commonly characterized by the presence of painless, bilateral swelling of the preauricular areas?**

- A. Parotid gland neoplasm

- B. Obstructive sialadenitis

- C. Mumps

- D. Sjögren's syndrome

- E. Benign lymphoepithelial lesion

- **Correct Answer**: D. Sjögren's syndrome

- **Explanation**: Sjögren’s syndrome features autoimmune-induced chronic, painless, and bilateral swelling of the salivary glands, particularly the preauricular parotids.

### Question 26

26. **A 50-year-old male presents with difficulty swallowing and severe tooth sensitivity. Oral examination reveals erythematous and smooth tongue lesions. What dietary deficiency might be causing these symptoms?**

- A. Vitamin A

- B. Vitamin C

- C. Vitamin D

- D. Vitamin B12

- E. Calcium

- **Correct Answer**: D. Vitamin B12

- **Explanation**: Deficiency in Vitamin B12 can result in glossitis and related symptoms like erythematous, smooth tongue lesions aside from systemic manifestations.

### Question 27

27. **Which of the following is the primary concern in the management of a patient with a dislocated TMJ?**

- A. Reducing the dislocation manually

- B. Prescribing antibiotics

- C. Administering muscle relaxants

- D. Immobilizing the mandible for 6 weeks

- E. Performing immediate surgical intervention

- **Correct Answer**: A. Reducing the dislocation manually

- **Explanation**: The initial priority is to manually reduce the TMJ dislocation to restore normal joint function and relieve pain.

### Question 28

28. **A 35-year-old patient presents with a jaw swelling associated with an unerupted mandibular third molar. Radiographs show a well-defined radiolucent lesion with a sclerotic border. What is the most likely diagnosis?**

- A. Ameloblastoma

- B. Odontogenic keratocyst

- C. Dentigerous cyst

- D. Periapical cyst

- E. Traumatic bone cyst

- **Correct Answer**: C. Dentigerous cyst

- **Explanation**: A dentigerous cyst is typically associated with the crown of an unerupted tooth and presents as a well-defined radiolucent lesion with a sclerotic border.

### Question 29

29. **Which of the following is least likely to be associated with osteoradionecrosis of the jaw?**

- A. Traumatic extractions post-radiation therapy

- B. High-dose radiation therapy

- C. Poor oral hygiene

- D. Chemotherapy

- E. Mandibular location of previous radiation

- **Correct Answer**: D. Chemotherapy

- **Explanation**: Osteoradionecrosis is primarily linked to radiation therapy rather than chemotherapy. Radiation leads to compromised vascular supply and bone necrosis in the treated region.

### Question 30

30. **Which nerve block is most commonly used for pain relief during maxillary anterior teeth procedures?**

- A. Greater palatine nerve block

- B. Inferior alveolar nerve block

- C. Nasopalatine nerve block

- D. Middle superior alveolar nerve block

- E. Anterior superior alveolar nerve block

- **Correct Answer**: E. Anterior superior alveolar nerve block

- **Explanation**: The anterior superior alveolar nerve block effectively anesthetizes the maxillary anterior teeth, providing pain relief for procedures in this region.

### Question 31

31. **What is the most common type of malignant tumor found within the salivary glands?**

- A. Pleomorphic adenoma

- B. Adenoid cystic carcinoma

- C. Mucoepidermoid carcinoma

- D. Warthin tumor

- E. Acinic cell carcinoma

- **Correct Answer**: C. Mucoepidermoid carcinoma

- **Explanation**: Mucoepidermoid carcinoma is the most frequent malignant tumor within the salivary glands, particularly the parotid gland.

### Question 32

32. **In the management of mandibular fractures, which of the following is considered the gold standard for rigid fixation?**

- A. Arch bars

- B. Resorbable plates

- C. Non-resorbable plates and screws

- D. Intermaxillary fixation (IMF)

- E. External fixator

- **Correct Answer**: C. Non-resorbable plates and screws

- **Explanation**: Non-resorbable plates and screws are the gold standard for achieving rigid and stable fixation of mandibular fractures.

### Question 33

33. **Which condition is characterized by “onion skin” periosteal reaction on radiographs?**

- A. Osteosarcoma

- B. Ewing’s sarcoma

- C. Osteomyelitis

- D. Ameloblastoma

- E. Chondrosarcoma

- **Correct Answer**: B. Ewing’s sarcoma

- **Explanation**: Ewing’s sarcoma typically shows an “onion skin” appearance on radiographs, which indicates layers of periosteal reaction due to the aggressive tumor.

### Question 34

34. **A 40-year-old female presents with periodic episodes of jaw pain, clicking, and limited mouth opening. What is the most likely diagnosis?**

- A. Osteoarthritis of the TMJ

- B. Myofascial pain syndrome

- C. Ankylosis of the TMJ

- D. Internal derangement of the TMJ

- E. Rheumatoid arthritis of the TMJ

- **Correct Answer**: D. Internal derangement of the TMJ

- **Explanation**: Internal derangement involves issues like disc displacement within the TMJ and is characterized by symptoms such as pain, clicking, and limited jaw movement.

### Question 35

35. **Which of the following is a hallmark feature of fibrous dysplasia in the maxillofacial region?**

- A. Well-defined radiopaque lesion

- B. Ground-glass appearance on radiographs

- C. Multilocular radiolucency

- D. Sunburst pattern

- E. Cotton wool appearance

- **Correct Answer**: B. Ground-glass appearance on radiographs

- **Explanation**: Fibrous dysplasia typically presents a "ground-glass" appearance on radiographs, indicating the abnormal fibro-osseous tissue within the bone.

### Question 36

36. **A 30-year-old male presents with chronic non-healing ulcers on the oral mucosa. A biopsy reveals granulomas with multinucleated giant cells. What is the most likely diagnosis?**

- A. Tuberculosis

- B. Sarcoidosis

- C. Crohn’s disease

- D. Syphilis

- E. Histoplasmosis

- **Correct Answer**: B. Sarcoidosis

- **Explanation**: Sarcoidosis frequently presents with granulomas containing multinucleated giant cells, which can be identified in chronic non-healing ulcers of the oral mucosa.

### Question 37

37. **A patient with a history of bisphosphonate use presents with jaw pain and exposed bone. What is the likely diagnosis?**

- A. Osteomyelitis

- B. Osteonecrosis of the jaw

- C. Osteoradionecrosis

- D. Periapical abscess

- E. Dental caries

- **Correct Answer**: B. Osteonecrosis of the jaw

- **Explanation**: Bisphosphonate-associated osteonecrosis of the jaw is identified by the presence of exposed bone and pain, often in patients who have a history of bisphosphonate use.

### Question 38

38. **A child presents with dental trauma resulting in the avulsion of a primary incisor. What is the recommended management?**

- A. Replant the tooth immediately

- B. Place the tooth in milk and seek dental care

- C. Do not replant the tooth and schedule follow-up

- D. Perform a temporary splint

- E. Administer systemic antibiotics

- **Correct Answer**: C. Do not replant the tooth and schedule follow-up

- **Explanation**: Primary (baby) teeth should not be replanted as it can harm the developing permanent teeth. Follow-up with a dentist is essential to monitor for potential complications.

### Question 39

39. **In which syndrome is there a triad of cleft lip/palate, mandibular hypoplasia, and hypoplastic zygomas?**

- A. Crouzon syndrome

- B. Treacher Collins syndrome

- C. Pierre Robin syndrome

- D. Apert syndrome

- E. Goldenhar syndrome

- **Correct Answer**: B. Treacher Collins syndrome

- **Explanation**: Treacher Collins syndrome features these craniofacial abnormalities, including mandibular hypoplasia, cleft lip/palate, and underdeveloped zygomatic bones.

### Question 40

40. **Which type of cyst is most commonly associated with non-vital teeth?**

- A. Radicular cyst

- B. Dentigerous cyst

- C. Odontogenic keratocyst

- D. Nasopalatine duct cyst

- E. Globulomaxillary cyst

- **Correct Answer**: A. Radicular cyst

- **Explanation**: Radicular cysts form at the apex of non-vital teeth, often resulting from pulpal necrosis and chronic inflammation.

### Question 41

41. **Which factor is most predictive of the likelihood of a patient developing post-operative infections after orthognathic surgery?**

- A. Length of the surgery

- B. Patient's age

- C. Type of anesthesia used

- D. Pre-existing periodontal disease

- E. Gender of the patient

- **Correct Answer**: D. Pre-existing periodontal disease

- **Explanation**: Pre-existing periodontal disease elevates the risk of post-operative infections due to increased bacterial load and compromised oral health status.

### Question 42

42. **A 25-year-old patient complains of pain, swelling, and limited mouth opening a few days after a lower third molar extraction. What is the most likely diagnosis?**

- A. Osteomyelitis

- B. Alveolar osteitis (dry socket)

- C. Submasseteric abscess

- D. Temporomandibular joint dislocation

- E. Trismus

- **Correct Answer**: C. Submasseteric abscess

- **Explanation**: Submasseteric abscesses can form following a lower third molar extraction, leading to significant discomfort, swelling, and restricted mouth opening (trismus).

### Question 43

43. **Which syndrome is characterized by bilateral cleft lip and palate, absent upper central incisors, and mesiodens?**

- A. Crouzon syndrome

- B. Van der Woude syndrome

- C. Apert syndrome

- D. Pierre Robin syndrome

- E. Ellis-van Creveld syndrome

- **Correct Answer**: E. Ellis-van Creveld syndrome

- **Explanation**: Ellis-van Creveld syndrome presents congenital issues such as bilateral cleft lip and palate, missing upper central incisors, and mesiodens.

### Question 44

44. **A 50-year-old male presents with a painless, slow-growing mass in the hard palate. Biopsy shows pleomorphic adenoma. What is the appropriate management?**

- A. Observation

- B. Surgical excision with clear margins

- C. Radiation therapy

- D. Chemotherapy

- E. Cryotherapy

- **Correct Answer**: B. Surgical excision with clear margins

- **Explanation**: Complete surgical excision with clear margins is the treatment of choice for pleomorphic adenoma to prevent recurrence.

### Question 45

45. **Which imaging technique is most appropriate for evaluating the soft tissue extent of oral cancers?**

- A. Panoramic radiograph

- B. Cephalometric radiograph

- C. CT scan

- D. Ultrasound

- E. MRI

- **Correct Answer**: E. MRI

- **Explanation**: MRI provides superior soft tissue contrast, making it ideal for assessing the extent of soft tissue invasion in oral cancers.

### Question 46

46. **What is the primary reason for immobilizing the mandible in patients with condylar fractures?**

- A. To prevent infection

- B. To ensure proper alignment of the condyle

- C. To allow muscular healing

- D. To maintain nutrition

- E. To prevent TMJ ankylosis

- **Correct Answer**: B. To ensure proper alignment of the condyle

- **Explanation**: Immobilization aids in the correct alignment and proper healing of the condylar fractures, preserving occlusal function.

### Question 47

47. **A 55-year-old male presents with paresthesia of the lower lip and chin following a dental extraction. Which nerve is most likely affected?**

- A. Lingual nerve

- B. Inferior alveolar nerve

- C. Mental nerve

- D. Buccal nerve

- E. Facial nerve

- **Correct Answer**: C. Mental nerve

- **Explanation**: Damage to the mental nerve, a branch of the inferior alveolar nerve, can lead to numbness or paresthesia of the lower lip and chin.

### Question 48

48. **Which condition is diagnosed by the presence of linearly arranged multinucleated giant cells and fibrous stroma on a biopsy of a jaw lesion?**

- A. Ameloblastoma

- B. Odontogenic keratocyst

- C. Central giant cell granuloma

- D. Aneurysmal bone cyst

- E. Osteosarcoma

- **Correct Answer**: C. Central giant cell granuloma

- **Explanation**: Central giant cell granuloma is characterized by multinucleated giant cells within a fibrous matrix as seen on biopsy.

### Question 49

49. **In patients with advanced osteoradionecrosis of the mandible, what is the most definitive treatment?**

- A. Hyperbaric oxygen therapy

- B. Antibiotics

- C. Surgical resection and reconstruction

- D. Conservative debridement

- E. Bisphosphonate therapy

- **Correct Answer**: C. Surgical resection and reconstruction

- **Explanation**: Advanced cases of osteoradionecrosis often require surgical resection of necrotic bone and subsequent reconstruction for functional and aesthetic rehabilitation.

### Question 50

50. **Which genetic disorder is most commonly associated with multiple odontogenic keratocysts?**

- A. Marfan syndrome

- B. Ehlers-Danlos syndrome

- C. Gardner syndrome

- D. Gorlin syndrome (Nevoid Basal Cell Carcinoma Syndrome)

- E. Von Hippel-Lindau syndrome

- **Correct Answer**: D. Gorlin syndrome (Nevoid Basal Cell Carcinoma Syndrome)

- **Explanation**: Gorlin syndrome, or Nevoid Basal Cell Carcinoma Syndrome, typically features multiple odontogenic keratocysts along with other abnormalities such as basal cell carcinomas and skeletal defects.
