# Supplementary material for: How valuable are the questions and answers generated by large language models in oral and maxillofacial surgery?
Source: PLoS One. 2025 May 28;20(5):e0322529. doi: 10.1371/journal.pone.0322529 (PMC12119001; doi:10.1371/journal.pone.0322529)
Supplement: S1 File — (ZIP) [file pone.0322529.s001.zip › gpt4/gpt4-gpt4o.docx]

### 1. Simple Knowledge Measurement

1. **Which nerve is most commonly injured during a mandibular third molar extraction?**

- A. Facial nerve

- B. Hypoglossal nerve

- C. Inferior alveolar nerve

- D. Lingual nerve

- E. Mental nerve

- **Correct Answer:** C. Inferior alveolar nerve

- **Basis:** The inferior alveolar nerve runs through the mandibular canal and is most at risk of injury during lower third molar extractions due to its proximity to the surgical site.

2. **What is the most common cause of maxillofacial trauma?**

- A. Sports injuries

- B. Falls

- C. Motor vehicle accidents

- D. Assaults

- E. Work-related injuries

- **Correct Answer:** C. Motor vehicle accidents

- **Basis:** Motor vehicle accidents commonly result in high-impact injuries, making them the leading cause of maxillofacial trauma.

3. **Which muscle is primarily responsible for elevating the mandible?**

- A. Temporalis

- B. Masseter

- C. Medial pterygoid

- D. Lateral pterygoid

- E. Digastric

- **Correct Answer:** B. Masseter

- **Basis:** The masseter is a powerful masticatory muscle that primarily functions to elevate the mandible, facilitating the closing of the jaw.

### 2. Causal Inference

4. **What is the primary reason for performing a Le Fort I osteotomy?**

- A. To correct mandibular prognathism

- B. To address maxillary hypoplasia

- C. To repair orbital fractures

- D. To treat temporomandibular joint disorder

- E. To remove impacted third molars

- **Correct Answer:** B. To address maxillary hypoplasia

- **Basis:** Le Fort I osteotomy involves repositioning the maxilla and is frequently performed to correct maxillary hypoplasia, enhancing facial symmetry and occlusion.

5. **Why is antibiotic prophylaxis recommended before dental procedures in patients with a history of infective endocarditis?**

- A. To prevent tooth decay

- B. To reduce pain

- C. To prevent bacterial endocarditis

- D. To minimize swelling

- E. To improve wound healing

- **Correct Answer:** C. To prevent bacterial endocarditis

- **Basis:** In patients with a history of infective endocarditis, antibiotic prophylaxis is critical before dental procedures to prevent bacteremia that could lead to bacterial endocarditis.

### 3. Example through Patient Case

6. **A 45-year-old patient presents with limited mouth opening and pain near the ear. Which condition is most likely?**

- A. Temporomandibular joint disorder

- B. Oral cancer

- C. Impacted molar

- D. Salivary gland infection

- E. Maxillary sinusitis

- **Correct Answer:** A. Temporomandibular joint disorder

- **Basis:** Symptoms of limited mouth opening and pain near the ear typically point to temporomandibular joint disorders, which affect the joint that connects the jaw to the skull.

7. **A 30-year-old male presents with a swelling in the floor of the mouth that increases in size during meals. What is the likely diagnosis?**

- A. Sialolithiasis

- B. Oral candidiasis

- C. Mucocele

- D. Squamous cell carcinoma

- E. Ranula

- **Correct Answer:** A. Sialolithiasis

- **Basis:** Swelling that becomes more prominent during meals is indicative of sialolithiasis, which involves the formation of salivary stones that block saliva flow, causing gland swelling.

### 4. Includes Schematic Diagram, Photography, X ray, CT and/or MRI

8. **An X-ray shows a radiolucent lesion at the apex of a non-vital tooth. What is the most likely diagnosis?**

- A. Periapical abscess

- B. Ameloblastoma

- C. Dentigerous cyst

- D. Osteosarcoma

- E. Odontoma

- **Correct Answer:** A. Periapical abscess

- **Basis:** A radiolucent area at the apex of a non-vital tooth is commonly seen in periapical abscesses, which result from infection extending from the tooth's pulp.

9. **A CT scan reveals a "ground glass" appearance of the maxillary sinus. What condition does this suggest?**

- A. Acute sinusitis

- B. Chronic sinusitis

- C. Fibrous dysplasia

- D. Osteomyelitis

- E. Paget's disease

- **Correct Answer:** C. Fibrous dysplasia

- **Basis:** The "ground glass" appearance on imaging is characteristic of fibrous dysplasia, due to the abnormal fibrous tissue replacing normal bone.

### Continued Questions

10. **A patient presents with unilateral jaw pain, limited mouth opening, and a recent history of trauma. What imaging is most appropriate?**

- A. Panoramic radiograph

- B. MRI

- C. CT scan

- D. Occlusal radiograph

- E. Lateral cephalogram

- **Correct Answer:** C. CT scan

- **Basis:** CT scans provide detailed cross-sectional images and are ideal for assessing bone fractures and complex anatomical structures following trauma.

11. **Which artery is the primary blood supply to the mandible?**

- A. Lingual artery

- B. Inferior alveolar artery

- C. Facial artery

- D. Maxillary artery

- E. Superior alveolar artery

- **Correct Answer:** B. Inferior alveolar artery

- **Basis:** The inferior alveolar artery, a branch of the maxillary artery, supplies the mandible and its dental structures.

12. **What is the primary treatment for an ameloblastoma?**

- A. Chemotherapy

- B. Radiation therapy

- C. Enucleation and curettage

- D. Wide local excision

- E. Observation

- **Correct Answer:** D. Wide local excision

- **Basis:** Due to its locally aggressive nature, the primary treatment for ameloblastoma involves wide local excision to minimize the risk of recurrence.

13. **A panoramic radiograph shows a well-defined, unilocular radiolucency associated with an unerupted third molar. What is the most likely diagnosis?**

- A. Ameloblastoma

- B. Dentigerous cyst

- C. Keratocystic odontogenic tumor

- D. Periapical cyst

- E. Osteosarcoma

- **Correct Answer:** B. Dentigerous cyst

- **Basis:** Dentigerous cysts commonly form around the crowns of unerupted teeth, presenting as well-defined radiolucent lesions.

14. **What is the most common benign salivary gland tumor?**

- A. Mucoepidermoid carcinoma

- B. Adenoid cystic carcinoma

- C. Pleomorphic adenoma

- D. Warthin tumor

- E. Acinic cell carcinoma

- **Correct Answer:** C. Pleomorphic adenoma

- **Basis:** Pleomorphic adenoma is the most common benign tumor of the salivary glands and often affects the parotid gland.

15. **Which muscle assists in the retraction of the mandible?**

- A. Masseter

- B. Medial pterygoid

- C. Lateral pterygoid

- D. Temporalis

- E. Buccinator

- **Correct Answer:** D. Temporalis

- **Basis:** The posterior fibers of the temporalis muscle aid in retracting the mandible.

16. **In what situation is an orthognathic surgery typically indicated?**

- A. Impacted third molars

- B. Severe obstructive sleep apnea

- C. Minor malocclusion

- D. Temporomandibular joint dysfunction

- E. Periapical abscess

- **Correct Answer:** B. Severe obstructive sleep apnea

- **Basis:** Orthognathic surgery is indicated for severe obstructive sleep apnea to reposition the jaws and improve airway dimensions.

17. **A CT scan shows a "sunburst" pattern in the jaw. What condition does this suggest?**

- A. Ameloblastoma

- B. Osteosarcoma

- C. Fibrous dysplasia

- D. Osteomyelitis

- E. Chondrosarcoma

- **Correct Answer:** B. Osteosarcoma

- **Basis:** The "sunburst" pattern on imaging is indicative of osteosarcoma, due to the spiculated new bone formation.

18. **A panoramic radiograph shows bilateral radiopaque areas in the mandibular angle region. What is the likely diagnosis?**

- A. Sialolithiasis

- B. Tori mandibularis

- C. Odontoma

- D. Osteoma

- E. Ameloblastoma

- **Correct Answer:** B. Tori mandibularis

- **Basis:** Tori mandibularis are benign bony outgrowths commonly found bilaterally in the mandibular region.

19. **A 60-year-old male presents with a painful, non-healing ulcer on the lateral border of the tongue. What is the most likely diagnosis?**

- A. Squamous cell carcinoma

- B. Oral lichen planus

- C. Aphthous ulcer

- D. Herpes simplex infection

- E. Traumatic ulcer

- **Correct Answer:** A. Squamous cell carcinoma

- **Basis:** Non-healing ulcers in the oral cavity, especially in older adults, are highly suggestive of squamous cell carcinoma, the most common oral malignancy.

20. **What is the most common complication following a mandibular fracture?**

- A. Infection

- B. Malocclusion

- C. Nerve injury

- D. Nonunion

- E. TMJ dysfunction

- **Correct Answer:** B. Malocclusion

- **Basis:** Malocclusion is a frequent complication due to potential misalignment of the mandible following a fracture.

### Additional Questions

21. **Which imaging modality is best for evaluating soft tissue structures of the temporomandibular joint?**

- A. Panoramic radiograph

- B. MRI

- C. CT scan

- D. Cone beam CT

- E. Ultrasound

- **Correct Answer:** B. MRI

- **Basis:** MRI provides superior soft tissue contrast, making it the best choice for evaluating the temporomandibular joint (TMJ) and its associated soft tissues, including the articular disc and surrounding ligaments.

22. **A patient presents with trismus, fever, and swelling of the submandibular space. What is the most likely diagnosis?**

- A. Ludwig's angina

- B. Pericoronitis

- C. Sialadenitis

- D. Mumps

- E. Temporomandibular joint disorder

- **Correct Answer:** A. Ludwig's angina

- **Basis:** Ludwig's angina is a severe bacterial infection involving the submandibular space, characterized by trismus (limited jaw opening), fever, and swelling, which can quickly compromise the airway.

23. **What is the gold standard treatment for a non-displaced mandibular condyle fracture?**

- A. Open reduction and internal fixation

- B. Closed reduction

- C. Conservative management with a soft diet

- D. External fixation

- E. Physiotherapy

- **Correct Answer:** C. Conservative management with a soft diet

- **Basis:** Non-displaced mandibular condyle fractures are typically treated conservatively to allow natural healing, which involves managing with a soft diet, limited jaw movement, and careful monitoring.

24. **A patient complains of persistent numbness in the lower lip after wisdom tooth extraction. Which nerve is likely affected?**

- A. Lingual nerve

- B. Inferior alveolar nerve

- C. Mental nerve

- D. Buccal nerve

- E. Glossopharyngeal nerve

- **Correct Answer:** C. Mental nerve

- **Basis:** The mental nerve, a branch of the inferior alveolar nerve, provides sensation to the lower lip, and injury to this nerve during or after extraction can result in persistent numbness in the affected area.

25. **A panoramic radiograph reveals a mixed radiolucent-radiopaque lesion in the posterior mandible. What is the likely diagnosis?**

- A. Ameloblastoma

- B. Ossifying fibroma

- C. Odontogenic keratocyst

- D. Fibrous dysplasia

- E. Cemento-osseous dysplasia

- **Correct Answer:** E. Cemento-osseous dysplasia

- **Basis:** Cemento-osseous dysplasia typically presents as a mixed radiolucent-radiopaque lesion in the jaw and is commonly located in the posterior mandible.

26. **Which condition is characterized by the triad of craniofacial dysostosis, syndactyly, and mental retardation?**

- A. Crouzon syndrome

- B. Apert syndrome

- C. Treacher Collins syndrome

- D. Pierre Robin sequence

- E. Goldenhar syndrome

- **Correct Answer:** B. Apert syndrome

- **Basis:** Apert syndrome prominently features craniofacial dysostosis (abnormal skull formation), syndactyly (fused fingers/toes), and varying degrees of mental retardation.

27. **What is the typical presentation of osteoradionecrosis of the jaw?**

- A. Painful, non-healing ulcer

- B. Radiopaque mass

- C. Soft tissue swelling

- D. Fistula formation

- E. All of the above

- **Correct Answer:** E. All of the above

- **Basis:** Osteoradionecrosis (ORN) of the jaw can present with a variety of symptoms including painful non-healing ulcers, radiographic changes, soft tissue swelling, and fistula formation.

28. **Which medication is commonly associated with medication-related osteonecrosis of the jaw (MRONJ)?**

- A. Penicillin

- B. Aspirin

- C. Bisphosphonates

- D. Antihistamines

- E. Steroids

- **Correct Answer:** C. Bisphosphonates

- **Basis:** Bisphosphonates, often prescribed for osteoporosis and metastatic bone disease, are strongly associated with the development of medication-related osteonecrosis of the jaw (MRONJ).

29. **Which cranial nerve is primarily responsible for sensation in the face?**

- A. Facial nerve

- B. Trigeminal nerve

- C. Hypoglossal nerve

- D. Glossopharyngeal nerve

- E. Vagus nerve

- **Correct Answer:** B. Trigeminal nerve

- **Basis:** The trigeminal nerve (cranial nerve V) is the main sensory nerve of the face, distributing to areas including the forehead, cheeks, and jaw.

30. **A 55-year-old patient presents with an asymptomatic swelling in the hard palate that has been gradually increasing in size. What is the most likely diagnosis?**

- A. Mucocele

- B. Pleomorphic adenoma

- C. Squamous cell carcinoma

- D. Torus palatinus

- E. Salivary gland cyst

- **Correct Answer:** D. Torus palatinus

- **Basis:** Torus palatinus is a benign, bony, hard, and slow-growing mass commonly found in the midline of the hard palate.

31. **Which condition is most likely to present with "onion skin" periosteal reaction on radiographs?**

- A. Osteosarcoma

- B. Ewing's sarcoma

- C. Osteomyelitis

- D. Chondrosarcoma

- E. Fibrous dysplasia

- **Correct Answer:** B. Ewing's sarcoma

- **Basis:** Ewing's sarcoma often exhibits an "onion skin" periosteal reaction due to layered bone formation as the tumor grows.

32. **A panoramic radiograph shows multiple radiolucent lesions in the mandible resembling soap bubbles. What is the likely diagnosis?**

- A. Ameloblastoma

- B. Odontogenic keratocyst

- C. Fibrous dysplasia

- D. Central giant cell granuloma

- E. Multiple myeloma

- **Correct Answer:** A. Ameloblastoma

- **Basis:** Ameloblastoma typically presents as a multilocular radiolucent lesion with a soap bubble or honeycomb appearance on radiographs.

33. **Which condition is characterized by "cotton wool" appearance on radiographs?**

- A. Osteomyelitis

- B. Fibrous dysplasia

- C. Paget's disease

- D. Osteosarcoma

- E. Amelogenesis imperfecta

- **Correct Answer:** C. Paget's disease

- **Basis:** Paget's disease of bone shows a distinctive "cotton wool" appearance on radiographs due to disorganized bone remodeling.

34. **A patient with a history of multiple dental extractions presents with a non-healing ulcer and bone exposure in the mandible. What is the likely diagnosis?**

- A. Osteoradionecrosis

- B. MRONJ

- C. Osteomyelitis

- D. Squamous cell carcinoma

- E. Ameloblastoma

- **Correct Answer:** B. MRONJ

- **Basis:** Medication-related osteonecrosis of the jaw (MRONJ) manifests as non-healing ulcers and exposed bone, often following dental extractions.

35. **Which condition is associated with "ground glass" appearance on radiographs?**

- A. Osteosarcoma

- B. Fibrous dysplasia

- C. Osteomyelitis

- D. Ameloblastoma

- E. Paget's disease

- **Correct Answer:** B. Fibrous dysplasia

- **Basis:** Fibrous dysplasia presents with a "ground glass" appearance on radiographs due to the presence of abnormal fibrous tissue.

36. **A patient presents with a painless, slow-growing mass in the parotid gland. What is the most likely diagnosis?**

- A. Pleomorphic adenoma

- B. Mucoepidermoid carcinoma

- C. Warthin tumor

- D. Adenoid cystic carcinoma

- E. Acinic cell carcinoma

- **Correct Answer:** A. Pleomorphic adenoma

- **Basis:** Pleomorphic adenoma is the most common benign tumor of the parotid gland, presenting as a painless, slow-growing mass.

37. **Which anatomical structure is at risk of injury during a submandibular gland excision?**

- A. Facial artery

- B. Inferior alveolar nerve

- C. Hypoglossal nerve

- D. Lingual nerve

- E. External carotid artery

- **Correct Answer:** D. Lingual nerve

- **Basis:** The lingual nerve is in close proximity to the submandibular gland and is at risk of injury during gland excision surgery.

38. **What is the first-line treatment for a patient presenting with a deep neck space infection?**

- A. Observation

- B. Oral antibiotics

- C. Intravenous antibiotics

- D. Surgical drainage

- E. Corticosteroids

- **Correct Answer:** C. Intravenous antibiotics

- **Basis:** Deep neck space infections are serious and require immediate treatment with intravenous antibiotics to prevent systemic spread and potential airway compromise.

39. **Which condition is characterized by the presence of "floating teeth" on radiographs?**

- A. Langerhans cell histiocytosis

- B. Ameloblastoma

- C. Fibrous dysplasia

- D. Osteomyelitis

- E. Paget's disease

- **Correct Answer:** A. Langerhans cell histiocytosis

- **Basis:** Langerhans cell histiocytosis can cause bony destruction, giving the appearance of "floating teeth" on radiographs.

40. **A 40-year-old patient presents with pain and swelling in the lower jaw after a recent tooth extraction. What is the most likely diagnosis?**

- A. Dry socket (alveolar osteitis)

- B. Osteomyelitis

- C. Ameloblastoma

- D. Odontogenic keratocyst

- E. Squamous cell carcinoma

- **Correct Answer:** A. Dry socket (alveolar osteitis)

- **Basis:** Pain occurring a few days after tooth extraction, along with the absence of a visible blood clot in the socket, is indicative of dry socket.

41. **What is the primary concern in a patient with bilateral mandibular fractures?**

- A. Airway obstruction

- B. Malocclusion

- C. Infection

- D. TMJ dysfunction

- E. Nerve injury

- **Correct Answer:** A. Airway obstruction

- **Basis:** Bilateral mandibular fractures pose a significant risk of airway obstruction due to potential displacement of bone fragments and resultant instability.

42. **A panoramic radiograph shows a multilocular radiolucent lesion in the maxilla with displacement of adjacent teeth. What is the likely diagnosis?**

- A. Ameloblastoma

- B. Odontogenic myxoma

- C. Central giant cell granuloma

- D. Ossifying fibroma

- E. Dentigerous cyst

- **Correct Answer:** B. Odontogenic myxoma

- **Basis:** Odontogenic myxomas appear as multilocular radiolucent lesions that often cause displacement of adjacent teeth due to their infiltrative growth pattern.

43. **Which of the following is a potential complication of untreated maxillary sinusitis?**

- A. Orbital cellulitis

- B. Meningitis

- C. Brain abscess

- D. Osteomyelitis of the maxilla

- E. All of the above

- **Correct Answer:** E. All of the above

- **Basis:** Untreated maxillary sinusitis can lead to severe complications such as orbital cellulitis, meningitis, brain abscess, and osteomyelitis of the maxilla due to the anatomical closeness of these structures.

44. **What is the most common cause of mandibular prognathism?**

- A. Genetic factors

- B. Trauma

- C. Endocrine disorders

- D. Nutritional deficiencies

- E. Environmental factors

- **Correct Answer:** A. Genetic factors

- **Basis:** Mandibular prognathism is primarily inherited and often results from genetic predisposition to skeletal abnormalities that cause an enlarged mandible.

45. **A patient presents with a painless, firm, and immobile mass in the anterior floor of the mouth. What is the most likely diagnosis?**

- A. Ranula

- B. Dermoid cyst

- C. Mucocele

- D. Salivary gland tumor

- E. Squamous cell carcinoma

- **Correct Answer:** B. Dermoid cyst

- **Basis:** Dermoid cysts typically present as painless, firm, and immobile masses located in the midline of the floor of the mouth.

46. **Which imaging modality is best for evaluating the extent of a salivary gland tumor?**

- A. Ultrasound

- B. MRI

- C. CT scan

- D. Sialography

- E. Panoramic radiograph

- **Correct Answer:** B. MRI

- **Basis:** MRI provides excellent soft tissue contrast, making it the most suitable imaging modality for evaluating the extent and nature of salivary gland tumors.

47. **A 50-year-old male presents with difficulty swallowing, hoarseness, and a mass in the neck. What is the most likely diagnosis?**

- A. Thyroid cancer

- B. Laryngeal cancer

- C. Parotid gland tumor

- D. Submandibular gland infection

- E. Cervical lymphadenopathy

- **Correct Answer:** B. Laryngeal cancer

- **Basis:** Symptoms of difficulty swallowing, hoarseness, and a neck mass are typical presentations of laryngeal cancer.

48. **A patient presents with a radiopaque lesion in the mandible that shows a "sunburst" pattern on imaging. What is the likely diagnosis?**

- A. Osteosarcoma

- B. Osteomyelitis

- C. Ameloblastoma

- D. Ossifying fibroma

- E. Fibrous dysplasia

- **Correct Answer:** A. Osteosarcoma

- **Basis:** The "sunburst" pattern is characteristic of osteosarcoma and is due to the aggressive periosteal reaction and new bone formation.

49. **What is the most common site for mandibular fractures?**

- A. Condyle

- B. Angle

- C. Body

- D. Symphysis

- E. Ramus

- **Correct Answer:** A. Condyle

- **Basis:** The condyle is the most common site of fracture in the mandible, likely due to its anatomical position and the leverage forces applied during trauma.

50. **A patient presents with chronic sinusitis and a radiopaque mass in the maxillary sinus on CT scan. What is the most likely diagnosis?**

- A. Mucocele

- B. Sinusitis

- C. Odontogenic cyst

- D. Osteoma

- E. Ameloblastoma

- **Correct Answer:** D. Osteoma

- **Basis:** Osteomas are benign bone tumors that can present as radiopaque masses in the maxillary sinus, often associated with symptoms of chronic sinusitis.
