# Supplementary material for: How valuable are the questions and answers generated by large language models in oral and maxillofacial surgery?
Source: PLoS One. 2025 May 28;20(5):e0322529. doi: 10.1371/journal.pone.0322529 (PMC12119001; doi:10.1371/journal.pone.0322529)
Supplement: S1 File — (ZIP) [file pone.0322529.s001.zip › gpt4o/gpt4o-CO.docx]

**1. Which of the following is the most common site for oral cancer?**

a) Buccal mucosa

b) Floor of the mouth

c) Hard palate

d) Tongue

e) Gingiva

**Answer:** d) Tongue

**Explanation:** Oral cancer most frequently develops on the tongue, particularly the sides and base, due to constant exposure to irritants such as tobacco and alcohol.

---

**2. A 45-year-old male presents with a painless, firm, and fixed lymph node in the submandibular region. What is the most likely diagnosis?**

a) Reactive lymphadenitis

b) Sialadenitis

c) Lymphoma

d) Metastatic oral cancer

e) Lipoma

**Answer:** d) Metastatic oral cancer

**Explanation:** The presence of a firm and fixed lymph node is highly indicative of metastatic spread, often from primary oral cancers.

---

**3. Which of the following is the most common type of odontogenic tumor?**

a) Ameloblastoma

b) Odontoma

c) Odontogenic myxoma

d) Adenomatoid odontogenic tumor

e) Calcifying epithelial odontogenic tumor

**Answer:** b) Odontoma

**Explanation:** Odontomas are the most prevalent odontogenic tumors and are considered developmental anomalies, often composed of dental tissues.

---

**4. A patient presents with a unilateral, painful swelling in the preauricular region that is exacerbated by eating. What is the most likely diagnosis?**

a) Mumps

b) Sialolithiasis

c) Sjögren's syndrome

d) Parotid gland tumor

e) Temporomandibular joint disorder

**Answer:** b) Sialolithiasis

**Explanation:** Pain and swelling in the preauricular region that worsen with eating typically suggest a blockage in the salivary duct, most commonly due to sialoliths.

---

**5. Which of the following is the most common site for maxillofacial fractures?**

a) Nasal bones

b) Zygoma

c) Mandible

d) Orbital floor

e) Maxilla

**Answer:** c) Mandible

**Explanation:** The mandible is frequently fractured due to its prominence and exposure to impact during trauma.

---

**6. A patient presents with a painless, slow-growing mass in the parotid gland. Which of the following is the most likely diagnosis?**

a) Pleomorphic adenoma

b) Warthin's tumor

c) Mucoepidermoid carcinoma

d) Adenoid cystic carcinoma

e) Acinic cell carcinoma

**Answer:** a) Pleomorphic adenoma

**Explanation:** Pleomorphic adenomas are the most common benign salivary gland tumors, typically presenting as painless, slow-growing masses.

---

**7. Which of the following nerves is most commonly injured during third molar extraction?**

a) Facial nerve

b) Glossopharyngeal nerve

c) Hypoglossal nerve

d) Inferior alveolar nerve

e) Lingual nerve

**Answer:** d) Inferior alveolar nerve

**Explanation:** Inferior alveolar nerve injury is a common complication of third molar extraction due to its proximity to the surgical site.

---

**8. A patient presents with a unilateral, painful, erythematous swelling in the submandibular region. Which of the following is the most likely diagnosis?**

a) Submandibular sialolithiasis

b) Submandibular sialadenitis

c) Submandibular gland tumor

d) Cervical lymphadenitis

e) Ranula

**Answer:** b) Submandibular sialadenitis

**Explanation:** Infection of the submandibular gland, often exacerbated by eating due to salivary flow blockages, presents with pain and erythema.

---

**9. Which of the following is the most common type of ameloblastoma?**

a) Unicystic ameloblastoma

b) Solid/multicystic ameloblastoma

c) Peripheral ameloblastoma

d) Desmoplastic ameloblastoma

e) Malignant ameloblastoma

**Answer:** b) Solid/multicystic ameloblastoma

**Explanation:** The solid/multicystic variant is the most common and aggressive form of ameloblastoma, noted for its potential to recur.

---

**10. A patient presents with a painful, non-healing ulcer on the lateral border of the tongue that has been present for 6 weeks. Which of the following is the most appropriate next step in management?**

a) Prescribe antibiotics

b) Perform an incisional biopsy

c) Refer for CT scan

d) Prescribe topical corticosteroids

e) Reassure and observe

**Answer:** b) Perform an incisional biopsy

**Explanation:** A persistent ulcer on the tongue, especially with risk factors such as tobacco use, necessitates a biopsy to rule out malignancy.

Absolutely! Here are the remaining questions with new answers and explanations:

---

**11. Which of the following is the most common site for osteonecrosis of the jaw associated with bisphosphonate therapy?**

a) Maxilla

b) Mandible

c) Zygoma

d) Nasal bones

e) Frontal bone

**Answer:** b) Mandible

**Explanation:** The mandible is more commonly affected by osteonecrosis related to bisphosphonate therapy due to its dense bone and lesser blood supply compared to the maxilla.

---

**12. A patient presents with a rapidly growing, painful swelling in the mandible. Radiographic examination reveals a multilocular radiolucency with irregular borders. Which of the following is the most likely diagnosis?**

a) Ameloblastoma

b) Odontogenic keratocyst

c) Central giant cell granuloma

d) Dentigerous cyst

e) Osteosarcoma

**Answer:** e) Osteosarcoma

**Explanation:** Rapidly growing and painful swelling with an irregular multilocular radiolucent lesion is suggestive of osteosarcoma, a malignant bone tumor.

---

**13. Which of the following is the most common type of maxillofacial trauma in pediatric patients?**

a) Mandibular fractures

b) Zygomatic fractures

c) Nasal fractures

d) Orbital fractures

e) Frontal bone fractures

**Answer:** c) Nasal fractures

**Explanation:** Nasal fractures are the most common facial injuries in children due to the prominence of the nasal bones.

---

**14. A patient presents with a painless, slow-growing mass in the submandibular gland. Fine needle aspiration biopsy reveals a mixture of epithelial and myoepithelial cells. Which of the following is the most likely diagnosis?**

a) Pleomorphic adenoma

b) Warthin's tumor

c) Mucoepidermoid carcinoma

d) Adenoid cystic carcinoma

e) Acinic cell carcinoma

**Answer:** a) Pleomorphic adenoma

**Explanation:** The presence of both epithelial and myoepithelial cells in a slow-growing mass is indicative of a pleomorphic adenoma, a common benign tumor in the submandibular gland.

---

**15. Which of the following is the most common site for cervical lymph node metastasis in oral squamous cell carcinoma?**

a) Level I (submandibular and submental nodes)

b) Level II (upper jugular nodes)

c) Level III (middle jugular nodes)

d) Level IV (lower jugular nodes)

e) Level V (posterior triangle nodes)

**Answer:** b) Level II (upper jugular nodes)

**Explanation:** Level II nodes, located at the upper jugular region, are a common site for metastasis from oral squamous cell carcinoma due to their proximity to the primary site.

---

**16. A patient presents with a unilateral, diffuse swelling of the parotid gland associated with dry mouth and dry eyes. Which of the following is the most likely diagnosis?**

a) Sjögren's syndrome

b) Sialolithiasis

c) Sialadenitis

d) Parotid gland tumor

e) Sarcoidosis

**Answer:** a) Sjögren's syndrome

**Explanation:** The combination of parotid gland swelling with xerostomia and keratoconjunctivitis sicca is typical of Sjögren's syndrome, an autoimmune condition.

---

**17. Which of the following is the most common type of odontogenic cyst?**

a) Radicular cyst

b) Dentigerous cyst

c) Odontogenic keratocyst

d) Lateral periodontal cyst

e) Glandular odontogenic cyst

**Answer:** a) Radicular cyst

**Explanation:** Radicular cysts are the most common odontogenic cysts, occurring at the apex of a non-vital tooth due to inflammation.

---

**18. A patient presents with a firm, painless mass in the floor of the mouth that causes elevation of the tongue. Which of the following is the most likely diagnosis?**

a) Ranula

b) Dermoid cyst

c) Lipoma

d) Pleomorphic adenoma

e) Mucoepidermoid carcinoma

**Answer:** b) Dermoid cyst

**Explanation:** A firm, painless mass in the floor of the mouth elevating the tongue is characteristic of a dermoid cyst, a developmental lesion.

---

**19. Which of the following is the most common site for osteoradionecrosis of the jaws?**

a) Maxilla

b) Mandible

c) Zygoma

d) Nasal bones

e) Frontal bone

**Answer:** b) Mandible

**Explanation:** The mandible is the most common site for osteoradionecrosis due to its less robust blood supply, making it more susceptible to damage from radiation therapy.

---

**20. A patient presents with a unilateral, fluctuant swelling in the buccal vestibule adjacent to a vital tooth. Radiographic examination reveals no abnormalities. Which of the following is the most likely diagnosis?**

a) Radicular cyst

b) Dentigerous cyst

c) Nasolabial cyst

d) Odontogenic keratocyst

e) Lateral periodontal cyst

**Answer:** c) Nasolabial cyst

**Explanation:** A fluctuant swelling in the buccal vestibule without radiographic abnormalities points toward a nasolabial cyst, a non-odontogenic cyst arising from nasolacrimal duct remnants.

---

**21. Which of the following is the most common site for a lingual thyroid?**

a) Anterior tongue

b) Posterior tongue

c) Lateral tongue

d) Floor of the mouth

e) Soft palate

**Answer:** b) Posterior tongue

**Explanation:** Lingual thyroid tissue most commonly presents at the base of the tongue, near the foramen cecum where the thyroid gland descended from during development.

---

**22. A patient presents with a rapidly growing, painless mass in the parotid gland. Fine needle aspiration biopsy reveals a predominance of lymphoid cells. Which of the following is the most likely diagnosis?**

a) Pleomorphic adenoma

b) Warthin's tumor

c) Mucoepidermoid carcinoma

d) Adenoid cystic carcinoma

e) Acinic cell carcinoma

**Answer:** b) Warthin's tumor

**Explanation:** A parotid mass with lymphoid cells on biopsy suggests Warthin's tumor, a benign cystadenoma that contains epithelial and lymphoid elements.

---

**23. Which of the following is the most common type of maxillofacial fracture in elderly patients?**

a) Mandibular fractures

b) Zygomatic fractures

c) Nasal fractures

d) Orbital fractures

e) Maxillary fractures

**Answer:** a) Mandibular fractures

**Explanation:** Elderly patients are prone to falls and have more brittle bones due to osteoporosis, making mandibular fractures more common.

---

**24. A patient presents with a slow-growing, painless mass in the submandibular gland. Fine needle aspiration biopsy reveals a predominance of uniform, basaloid cells with a cribriform pattern. Which of the following is the most likely diagnosis?**

a) Pleomorphic adenoma

b) Warthin's tumor

c) Mucoepidermoid carcinoma

d) Adenoid cystic carcinoma

e) Acinic cell carcinoma

**Answer:** d) Adenoid cystic carcinoma

**Explanation:** The presence of basaloid cells in a cribriform pattern strongly suggests adenoid cystic carcinoma, a malignant salivary gland tumor known for its perineural invasion.

---

**25. Which of the following is the most common site for a dentigerous cyst?**

a) Maxillary anterior region

b) Maxillary posterior region

c) Mandibular anterior region

d) Mandibular posterior region

e) Mandibular ramus

**Answer:** d) Mandibular posterior region

**Explanation:** Dentigerous cysts typically form around the crowns of unerupted or developing molars, specifically in the mandibular posterior region.

---

**26. A patient presents with a diffuse, bilateral swelling of the parotid glands associated with fever, malaise, and myalgia. Which of the following is the most likely diagnosis?**

a) Sjögren's syndrome

b) Sialolithiasis

c) Sialadenitis

d) Parotid gland tumor

e) Mumps

**Answer:** e) Mumps

**Explanation:** Bilateral parotid swelling combined with systemic symptoms like fever and myalgia are hallmark signs of mumps, a viral infection.

---

**27. Which of the following is the most common type of odontogenic tumor in children?**

a) Ameloblastoma

b) Odontoma

c) Odontogenic myxoma

d) Adenomatoid odontogenic tumor

e) Calcifying epithelial odontogenic tumor

**Answer:** b) Odontoma

**Explanation:** Odontomas are the most common odontogenic tumors in children, often discovered incidentally on radiographs.

---

**28. A patient presents with a painful, non-healing ulcer on the ventral surface of the tongue that has been present for 3 months. The patient has a history of heavy smoking and alcohol consumption. Which of the following is the most appropriate next step in management?**

a) Prescribe antibiotics

b) Perform an incisional biopsy

c) Refer for CT scan

d) Prescribe topical corticosteroids

e) Reassure and observe

**Answer:** b) Perform an incisional biopsy

**Explanation:** Non-healing ulcers in patients with risk factors for oral cancer warrant a biopsy to confirm or rule out malignancy.

---

**29. Which of the following is the most common site for a ranula?**

a) Buccal mucosa

b) Floor of the mouth

c) Hard palate

d) Tongue

e) Gingiva

**Answer:** b) Floor of the mouth

**Explanation:** A ranula, or mucocele of the major salivary glands, commonly occurs on the floor of the mouth due to accumulation of saliva from a ruptured salivary gland duct.

---

**30. A patient presents with a rapidly growing, painful mass in the mandible. Radiographic examination reveals a radiolucent lesion with irregular borders and a "sunburst" appearance. Which of the following is the most likely diagnosis?**

a) Ameloblastoma

b) Odontogenic keratocyst

c) Central giant cell granuloma

d) Dentigerous cyst

e) Osteosarcoma

**Answer:** e) Osteosarcoma

**Explanation:** Radiolucent lesions with a characteristic "sunburst" pattern and rapid growth indicate osteosarcoma, a primary malignant bone tumor.

---

**31. Which of the following is the most common type of maxillofacial trauma in adult patients?**

a) Mandibular fractures

b) Zygomatic fractures

c) Nasal fractures

d) Orbital fractures

e) Frontal bone fractures

**Answer:** a) Mandibular fractures

**Explanation:** Mandibular fractures are frequently encountered in adulthood due to their prominent position and susceptibility to trauma.

---

**32. A patient presents with a slow-growing, painless mass in the parotid gland. Fine needle aspiration biopsy reveals a predominance of mucous cells. Which of the following is the most likely diagnosis?**

a) Pleomorphic adenoma

b) Warthin's tumor

c) Mucoepidermoid carcinoma

d) Adenoid cystic carcinoma

e) Acinic cell carcinoma

**Answer:** c) Mucoepidermoid carcinoma

**Explanation:** Mucoepidermoid carcinoma typically consists of mucous-producing cells, indicating a malignant salivary gland tumor.

---

**33. Which of the following is the most common site for a nasopalatine duct cyst?**

a) Maxillary anterior region

b) Maxillary posterior region

c) Mandibular anterior region

d) Mandibular posterior region

e) Soft palate

**Answer:** a) Maxillary anterior region

**Explanation:** Nasopalatine duct cysts occur in the maxillary anterior region, near the incisive canal, resulting from remnants of the nasopalatine duct.

---

**34. A patient presents with a firm, painless mass in the tongue that has been slowly growing for 6 months. Which of the following is the most likely diagnosis?**

a) Squamous cell carcinoma

b) Fibroma

c) Lipoma

d) Hemangioma

e) Lymphangioma

**Answer:** b) Fibroma

**Explanation:** A slowly growing, firm, painless mass on the tongue is likely a fibroma, a benign fibrous growth resulting from chronic irritation.

---

**35. Which of the following is the most common type of odontogenic tumor in adults?**

a) Ameloblastoma

b) Odontoma

c) Odontogenic myxoma

d) Adenomatoid odontogenic tumor

e) Calcifying epithelial odontogenic tumor

**Answer:** a) Ameloblastoma

**Explanation:** Ameloblastomas are common benign odontogenic tumors in adults, characterized by their locally aggressive behavior.

---

**36. A patient presents with a unilateral, painful swelling in the submandibular region that is exacerbated by eating. Radiographic examination reveals a radiopaque mass within the submandibular duct. Which of the following is the most likely diagnosis?**

a) Submandibular sialolithiasis

b) Submandibular sialadenitis

c) Submandibular gland tumor

d) Cervical lymphadenitis

e) Ranula

**Answer:** a) Submandibular sialolithiasis

**Explanation:** Painful swelling in the submandibular region exacerbated by eating along with radiographic evidence of a stone suggests submandibular sialolithiasis.

---

**37. Which of the following is the most common site for a mucoepidermoid carcinoma?**

a) Parotid gland

b) Submandibular gland

c) Sublingual gland

d) Minor salivary glands of the palate

e) Minor salivary glands of the buccal mucosa

**Answer:** a) Parotid gland

**Explanation:** The parotid gland is the primary site for mucoepidermoid carcinoma, indicating it as the most common salivary gland malignancy.

---

**38. A patient presents with a painful, non-healing ulcer on the lateral border of the tongue that has been present for 4 weeks. The patient has a history of chewing tobacco. Which of the following is the most appropriate next step in management?**

a) Prescribe antibiotics

b) Perform an incisional biopsy

c) Refer for CT scan

d) Prescribe topical corticosteroids

e) Advise the patient to quit using tobacco products

**Answer:** b) Perform an incisional biopsy

**Explanation:** Persistent, non-healing ulcers with a history of tobacco use require a biopsy to assess for potential malignancy.

---

**39. Which of the following is the most common site for a pleomorphic adenoma?**

a) Parotid gland

b) Submandibular gland

c) Sublingual gland

d) Minor salivary glands of the palate

e) Minor salivary glands of the lip

**Answer:** a) Parotid gland

**Explanation:** Pleomorphic adenomas are most frequently found in the parotid gland, representing the most common benign salivary tumor.

---

**40. A patient presents with a rapidly growing, painless mass in the posterior mandible. Radiographic examination reveals a multilocular radiolucency with scalloped borders. Which of the following is the most likely diagnosis?**

a) Ameloblastoma

b) Odontogenic keratocyst

c) Central giant cell granuloma

d) Dentigerous cyst

e) Odontogenic myxoma

**Answer:** a) Ameloblastoma

**Explanation:** A multilocular radiolucency with scalloped borders in the posterior mandible is characteristic of ameloblastoma, known for its aggressive and recurrent behavior.

---

**41. Which of the following is the most common type of maxillofacial fracture resulting from motor vehicle accidents?**

a) Mandibular fractures

b) Zygomatic fractures

c) Nasal fractures

d) Orbital fractures

e) Le Fort fractures

**Answer:** e) Le Fort fractures

**Explanation:** Le Fort fractures are common in high-impact traumas, such as motor vehicle accidents, involving the midface.

---

**42. A patient presents with a slow-growing, painless mass in the parotid gland. Fine needle aspiration biopsy reveals a predominance of oncocytic cells. Which of the following is the most likely diagnosis?**

a) Pleomorphic adenoma

b) Warthin's tumor

c) Mucoepidermoid carcinoma

d) Adenoid cystic carcinoma

e) Acinic cell carcinoma

**Answer:** b) Warthin's tumor

**Explanation:** Oncocytic cells, featuring in a slow-growing parotid mass, are indicative of Warthin's tumor, a benign lesion.

---

**43. Which of the following is the most common site for a central giant cell granuloma?**

a) Maxillary anterior region

b) Maxillary posterior region

c) Mandibular anterior region

d) Mandibular posterior region

e) Mandibular condyle

**Answer:** d) Mandibular posterior region

**Explanation:** Central giant cell granulomas frequently localize in the mandibular posterior region, particularly in young individuals.

---

**44. A patient presents with a diffuse, bilateral enlargement of the parotid glands associated with xerostomia and rheumatoid arthritis. Which of the following is the most likely diagnosis?**

a) Sjögren's syndrome

b) Sialolithiasis

c) Sialadenitis

d) Parotid gland tumors

e) Sarcoidosis

**Answer:** a) Sjögren's syndrome

**Explanation:** Bilateral parotid enlargement, dry mouth, and association with autoimmune conditions like rheumatoid arthritis point towards Sjögren's syndrome.

---

**45. Which of the following is the most common type of odontogenic cyst associated with an impacted tooth?**

a) Radicular cyst

b) Dentigerous cyst

c) Odontogenic keratocyst

d) Lateral periodontal cyst

e) Glandular odontogenic cyst

**Answer:** b) Dentigerous cyst

**Explanation:** Dentigerous cysts, frequently associated with impacted teeth, develop around the crown of unerupted teeth.

**46. A patient presents with a painful, non-healing ulcer on the floor of the mouth that has been present for 2 months. The patient has a history of smoking and drinking alcohol. Which of the following is the most appropriate next step in management?**

a) Prescribe antibiotics

b) Perform an incisional biopsy

c) Refer for CT scan

d) Prescribe topical corticosteroids

e) Advise the patient to quit smoking and drinking alcohol

**Answer:** b) Perform an incisional biopsy

**Explanation:** A persistent ulcer in a high-risk individual necessitates an incisional biopsy to rule out or confirm malignancy, especially considering the patient's history of smoking and alcohol use.

---

**47. Which of the following is the most common site for a mucocele?**

a) Buccal mucosa

b) Floor of the mouth

c) Hard palate

d) Tongue

e) Lower lip

**Answer:** e) Lower lip

**Explanation:** Mucoceles most commonly occur on the lower lip due to their formation following trauma or blockage of the minor salivary gland ducts.

---

**48. A patient presents with a slow-growing, painless mass in the submandibular gland. Fine needle aspiration biopsy reveals a predominance of clear cells. Which of the following is the most likely diagnosis?**

a) Pleomorphic adenoma

b) Warthin's tumor

c) Mucoepidermoid carcinoma

d) Adenoid cystic carcinoma

e) Acinic cell carcinoma

**Answer:** e) Acinic cell carcinoma

**Explanation:** The presence of clear cells in a submandibular gland mass is suggestive of acinic cell carcinoma, which is a low-grade malignancy of the salivary glands.

---

**49. Which of the following is the most common site for a traumatic bone cyst?**

a) Maxillary anterior region

b) Maxillary posterior region

c) Mandibular anterior region

d) Mandibular posterior region

e) Mandibular condyle

**Answer:** d) Mandibular posterior region

**Explanation:** Traumatic bone cysts frequently occur in the mandibular posterior region, often identified incidentally on radiographs.

---

**50. A patient presents with a rapidly growing, painful mass in the maxillary sinus. Radiographic examination reveals a radiopaque lesion with a "ground glass" appearance. Which of the following is the most likely diagnosis?**

a) Osteoma

b) Fibrous dysplasia

c) Ossifying fibroma

d) Osteosarcoma

e) Chondrosarcoma

**Answer:** b) Fibrous dysplasia

**Explanation:** A "ground glass" radiographic appearance in a rapidly growing maxillary sinus lesion is characteristic of fibrous dysplasia, a benign fibro-osseous condition.
