# Supplementary material for: How valuable are the questions and answers generated by large language models in oral and maxillofacial surgery?
Source: PLoS One. 2025 May 28;20(5):e0322529. doi: 10.1371/journal.pone.0322529 (PMC12119001; doi:10.1371/journal.pone.0322529)
Supplement: S1 File — (ZIP) [file pone.0322529.s001.zip › gpt4o/gpt4o-gpt4.docx]

### Question 1

1. **Which nerve is most commonly injured during a mandibular third molar extraction?**

- **Answer**: C. Inferior alveolar nerve

- **Explanation**: The inferior alveolar nerve runs very close to the roots of the mandibular third molar and is the most commonly injured nerve during the extraction of these teeth.

### Question 2

2. **A 45-year-old male presents with a fractured zygomatic arch. Which imaging modality is most suitable to evaluate the fracture?**

- **Answer**: D. CT scan

- **Explanation**: CT scans are ideal for detailed visualization of bone structures, making them the best option for assessing zygomatic arch fractures.

### Question 3

3. **What is the most common cause of osteonecrosis of the jaw?**

- **Answer**: B. Bisphosphonate therapy

- **Explanation**: Bisphosphonates, which are used to treat osteoporosis and other bone diseases, have a strong association with osteonecrosis of the jaw.

### Question 4

4. **During TMJ surgical procedures, which structure is most at risk of injury?**

- **Answer**: B. Facial nerve

- **Explanation**: The facial nerve runs in close proximity to the TMJ and is at significant risk of injury during surgical procedures involving this joint.

### Question 5

5. **What is the first-line surgical intervention for severe obstructive sleep apnea unresponsive to CPAP therapy?**

- **Answer**: C. Uvulopalatopharyngoplasty (UPPP)

- **Explanation**: UPPP is a commonly performed procedure to remove excess tissue from the throat to widen the airway and is often effective for treating obstructive sleep apnea.

### Question 6

6. **What is the typical imaging feature of a dentigerous cyst on a panoramic radiograph?**

- **Answer**: B. Radiolucent area surrounding an unerupted tooth

- **Explanation**: Dentigerous cysts typically present as a well-defined radiolucent area that surrounds the crown of an unerupted tooth.

### Question 7

7. **In the case of a mandibular angle fracture, which of the following is the most crucial factor in deciding the treatment plan?**

- **Answer**: B. Degree of displacement

- **Explanation**: The degree of displacement is a crucial factor in deciding whether a mandibular angle fracture requires surgical intervention or can be managed conservatively.

### Question 8

8. **A 25-year-old female presents with a swelling at the angle of the mandible. On imaging, a well-circumscribed, radiolucent lesion with a “soap-bubble” appearance is identified. What is the most likely diagnosis?**

- **Answer**: A. Ameloblastoma

- **Explanation**: Ameloblastomas are typically benign but locally aggressive tumors that present with a "soap-bubble" or multiloculated radiolucent appearance on imaging.

### Question 9

9. **What surgical technique is typically used to correct mandibular prognathism?**

- **Answer**: B. Bilateral sagittal split osteotomy (BSSO)

- **Explanation**: BSSO is a common and effective surgical method used to correct mandibular prognathism by repositioning the jaw.

### Question 10

10. **In the setting of facial trauma, which Le Fort fracture type involves separation of the midface from the cranial base?**

- **Answer**: C. Le Fort III

- **Explanation**: Le Fort III fractures involve a horizontal separation of the midface from the cranial base and are often referred to as craniofacial disjunctions.

### Question 11

11. **A 60-year-old patient presents with an oral squamous cell carcinoma involving the floor of the mouth. Which of the following is the most appropriate initial imaging modality for staging the tumor?**

- **Answer**: D. Contrast-enhanced CT scan

- **Explanation**: Contrast-enhanced CT scans are essential for assessing the size, local invasion, and regional lymph node involvement of oral squamous cell carcinoma.

### Question 12

12. **Which condition is characterized by bilateral enlargement of the parotid glands and is often associated with chronic alcohol abuse?**

- **Answer**: E. Sialadenosis

- **Explanation**: Sialadenosis involves non-inflammatory, non-neoplastic enlargement of the salivary glands, frequently associated with chronic alcohol use, diabetes, and malnutrition.

### Question 13

13. **What is the preferred surgical treatment for a benign tumor located at the angle of the mandible?**

- **Answer**: B. Marginal resection

- **Explanation**: Marginal resection involves removing the tumor with a margin of normal tissue to ensure complete excision while preserving mandibular function and structure.

### Question 14

14. **A patient presents with a suspected mandibular fracture. Which physical examination finding is most indicative of this diagnosis?**

- **Answer**: B. Anterior open bite

- **Explanation**: An anterior open bite, where the front teeth do not touch when the jaw is closed, is commonly indicative of a mandibular fracture.

### Question 15

15. **Which type of flap is commonly used for large defects in oral cancer reconstruction that require both soft tissue and bony reconstruction?**

- **Answer**: D. Fibula free flap

- **Explanation**: The fibula free flap provides both bony and soft tissue components, making it ideal for large reconstructive surgeries following oral cancer resection.

### Question 16

16. **Which of these statements about odontogenic keratocyst (OKC) is true?**

- **Answer**: B. It has a high recurrence rate after treatment.

- **Explanation**: Odontogenic keratocysts are known for their high recurrence rate after treatment due to their aggressive and infiltrative nature.

### Question 17

17. **In orthognathic surgery, which of the following is considered when planning for a mandibular setback?**

- **Answer**: B. Increase in mandibular angle

- **Explanation**: Adjusting the mandibular angle is a critical aspect when planning a mandibular setback surgery to ensure proper functional and aesthetic outcomes.

### Question 18

18. **Which characteristic feature is often seen in panoramic radiographs of patients with amelogenesis imperfecta?**

- **Answer**: B. Generalized thinning of enamel

- **Explanation**: Amelogenesis imperfecta typically presents with generalized thinning or absence of enamel, leading to a radiographic appearance where enamel is nearly indistinguishable from dentin.

### Question 19

19. **A 35-year-old male is involved in a road traffic accident and presents with a midface fracture. The clinical and radiological findings suggest a zygomaticomaxillary complex fracture. Which clinical sign is most likely to be present?**

- **Answer**: D. Inferior orbital rim step deformity

- **Explanation**: A palpable step deformity at the inferior orbital rim is a common clinical sign associated with zygomaticomaxillary complex fractures.

### Question 20

20. **What is the main advantage of using intraoperative navigation systems in maxillofacial surgery?**

- **Answer**: D. Increased precision in anatomical localization

- **Explanation**: Intraoperative navigation systems enhance the precision of anatomical localization during surgery, improving surgical accuracy and outcomes.

### Question 21

21. **Which muscle is most commonly involved in trismus following a mandibular third molar extraction?**

- **Answer**: C. Medial pterygoid

- **Explanation**: The medial pterygoid muscle, located near the mandibular third molar, is often affected following extraction, leading to trismus.

### Question 22

22. **Which of the following is a common sign of an orbital floor fracture (blowout fracture)?**

- **Answer**: C. Enophthalmos

- **Explanation**: Enophthalmos, characterized by the sinking of the eye into the orbit, is a common sign of an orbital floor (blowout) fracture due to the loss of orbital support.

### Question 23

23. **Which antibiotic is recommended as first-line therapy for odontogenic infections that are anaerobic in nature?**

- **Answer**: D. Metronidazole

- **Explanation**: Metronidazole is effective against anaerobic bacteria, which are commonly involved in dental infections.

### Question 24

24. **A panoramic radiograph of a patient reveals a radiopaque lesion with a radiolucent halo in the posterior mandible. What is the most likely diagnosis?**

- **Answer**: C. Odontoma

- **Explanation**: Odontomas often appear as radiopaque lesions with a surrounding radiolucent lesion, reflecting their mixed composition of dental hard tissues.

### Question 25

25. **Which condition is commonly characterized by the presence of painless, bilateral swelling of the preauricular areas?**

- **Answer**: D. Sjögren's syndrome

- **Explanation**: Sjögren's syndrome, an autoimmune disorder, can present with chronic, painless, bilateral swelling of the parotid glands.

### Question 26

26. **A 50-year-old male presents with difficulty swallowing and severe tooth sensitivity. Oral examination reveals erythematous and smooth tongue lesions. What dietary deficiency might be causing these symptoms?**

- **Answer**: D. Vitamin B12

- **Explanation**: Vitamin B12 deficiency can present with symptoms like glossitis (smooth, erythematous tongue) and other oral manifestations such as difficulty swallowing and tooth sensitivity.

### Question 27

27. **Which of the following is the primary concern in the management of a patient with a dislocated TMJ?**

- **Answer**: A. Reducing the dislocation manually

- **Explanation**: The immediate concern is to reduce the TMJ dislocation to restore function and relieve pain.

### Question 28

28. **A 35-year-old patient presents with a jaw swelling associated with an unerupted mandibular third molar. Radiographs show a well-defined radiolucent lesion with a sclerotic border. What is the most likely diagnosis?**

- **Answer**: C. Dentigerous cyst

- **Explanation**: Dentigerous cysts commonly present as well-defined radiolucent lesions around unerupted teeth, often with a sclerotic border.

### Question 29

29. **Which of the following is least likely to be associated with osteoradionecrosis of the jaw?**

- **Answer**: D. Chemotherapy

- **Explanation**: Osteoradionecrosis is more directly related to radiation therapy than chemotherapy, even though both may be used in cancer treatment.

### Question 30

30. **Which nerve block is most commonly used for pain relief during maxillary anterior teeth procedures?**

- **Answer**: E. Anterior superior alveolar nerve block

- **Explanation**: The anterior superior alveolar nerve block effectively anesthetizes the maxillary anterior teeth for dental procedures.

### Question 31

31. **What is the most common type of malignant tumor found within the salivary glands?**

- **Answer**: C. Mucoepidermoid carcinoma

- **Explanation**: Mucoepidermoid carcinoma is the most common malignant tumor of the salivary glands, especially in the parotid gland.

### Question 32

32. **In the management of mandibular fractures, which of the following is considered the gold standard for rigid fixation?**

- **Answer**: C. Non-resorbable plates and screws

- **Explanation**: Non-resorbable plates and screws provide the most reliable rigid fixation for mandibular fractures, ensuring stability and proper healing.

### Question 33

33. **Which condition is characterized by “onion skin” periosteal reaction on radiographs?**

- **Answer**: B. Ewing’s sarcoma

- **Explanation**: Ewing’s sarcoma typically presents with an “onion skin” periosteal reaction on radiographs due to the layered response of the periosteum to the tumor.

### Question 34

34. **A 40-year-old female presents with periodic episodes of jaw pain, clicking, and limited mouth opening. What is the most likely diagnosis?**

- **Answer**: D. Internal derangement of the TMJ

- **Explanation**: Internal derangement of the TMJ often involves displacement or dysfunction of the disc within the joint, leading to pain, clicking, and limited mouth opening.

### Question 35

35. **Which of the following is a hallmark feature of fibrous dysplasia in the maxillofacial region?**

- **Answer**: B. Ground-glass appearance on radiographs

- **Explanation**: Fibrous dysplasia presents with a “ground-glass” radiographic appearance due to the abnormal fibro-osseous tissue within the bone.

### Question 36

36. **A 30-year-old male presents with chronic non-healing ulcers on the oral mucosa. A biopsy reveals granulomas with multinucleated giant cells. What is the most likely diagnosis?**

- **Answer**: B. Sarcoidosis

- **Explanation**: Sarcoidosis commonly presents with granulomas containing multinucleated giant cells, which can manifest in the oral mucosa among other systemic sites.

### Question 37

37. **A patient with a history of bisphosphonate use presents with jaw pain and exposed bone. What is the likely diagnosis?**

- **Answer**: B. Osteonecrosis of the jaw

- **Explanation**: Bisphosphonate-Related Osteonecrosis of the Jaw (BRONJ) is characterized by exposed bone and pain in patients with a history of bisphosphonate use.

### Question 38

38. **A child presents with dental trauma resulting in the avulsion of a primary incisor. What is the recommended management?**

- **Answer**: C. Do not replant the tooth and schedule follow-up

- **Explanation**: Primary teeth should not be replanted because doing so can damage the developing permanent teeth beneath them. Instead, follow-up care is needed.

### Question 39

39. **In which syndrome is there a triad of cleft lip/palate, mandibular hypoplasia, and hypoplastic zygomas?**

- **Answer**: B. Treacher Collins syndrome

- **Explanation**: Treacher Collins syndrome is characterized by mandibular hypoplasia, cleft lip/palate, and underdeveloped zygomatic bones.

### Question 40

40. **Which type of cyst is most commonly associated with non-vital teeth?**

- **Answer**: A. Radicular cyst

- **Explanation**: Radicular cysts, also known as periapical cysts, are associated with the apex of non-vital teeth following pulpal necrosis.

### Question 41

41. **Which factor is most predictive of the likelihood of a patient developing post-operative infections after orthognathic surgery?**

- **Answer**: D. Pre-existing periodontal disease

- **Explanation**: Pre-existing periodontal disease increases the risk of post-operative infections due to the higher bacterial load in the oral cavity.

### Question 42

42. **A 25-year-old patient complains of pain, swelling, and limited mouth opening a few days after a lower third molar extraction. What is the most likely diagnosis?**

- **Answer**: C. Submasseteric abscess

- **Explanation**: A submasseteric abscess can form after a lower third molar extraction, presenting with pain, swelling, and limited mouth opening (trismus).

### Question 43

43. **Which syndrome is characterized by bilateral cleft lip and palate, absent upper central incisors, and mesiodens?**

- **Answer**: E. Ellis-van Creveld syndrome

- **Explanation**: Ellis-van Creveld syndrome includes congenital anomalies such as bilateral cleft lip/palate, absent upper central incisors, and mesiodens.

### Question 44

44. **A 50-year-old male presents with a painless, slow-growing mass in the hard palate. Biopsy shows pleomorphic adenoma. What is the appropriate management?**

- **Answer**: B. Surgical excision with clear margins

- **Explanation**: Surgical excision with clear margins is the primary treatment for pleomorphic adenoma to ensure complete removal and prevent recurrence.

### Question 45

45. **Which imaging technique is most appropriate for evaluating the soft tissue extent of oral cancers?**

- **Answer**: E. MRI

- **Explanation**: MRI provides superior soft tissue contrast, making it the best imaging modality for evaluating the extent of soft tissue involvement in oral cancers.

### Question 46

46. **What is the primary reason for immobilizing the mandible in patients with condylar fractures?**

- **Answer**: B. To ensure proper alignment of the condyle

- **Explanation**: Immobilization ensures that the condyle is properly aligned during healing to maintain normal jaw function.

### Question 47

47. **A 55-year-old male presents with paresthesia of the lower lip and chin following a dental extraction. Which nerve is most likely affected?**

- **Answer**: C. Mental nerve

- **Explanation**: The mental nerve, a branch of the inferior alveolar nerve, is commonly involved in paresthesia of the lower lip and chin following dental procedures.

### Question 48

48. **Which condition is diagnosed by the presence of linearly arranged multinucleated giant cells and fibrous stroma on a biopsy of a jaw lesion?**

- **Answer**: C. Central giant cell granuloma

- **Explanation**: Central giant cell granuloma is characterized by multinucleated giant cells within a fibrous stroma, commonly found in jaw lesions.

### Question 49

49. **In patients with advanced osteoradionecrosis of the mandible, what is the most definitive treatment?**

- **Answer**: C. Surgical resection and reconstruction

- **Explanation**: Advanced osteoradionecrosis typically necessitates surgical resection of affected bone followed by reconstruction to restore function and appearance.

### Question 50

50. **Which genetic disorder is most commonly associated with multiple odontogenic keratocysts?**

- **Answer**: D. Gorlin syndrome (Nevoid Basal Cell Carcinoma Syndrome)

- **Explanation**: Gorlin syndrome is associated with the presence of multiple odontogenic keratocysts as well as basal cell carcinomas.
