# Supplementary material for: How valuable are the questions and answers generated by large language models in oral and maxillofacial surgery?
Source: PLoS One. 2025 May 28;20(5):e0322529. doi: 10.1371/journal.pone.0322529 (PMC12119001; doi:10.1371/journal.pone.0322529)
Supplement: S1 File — (ZIP) [file pone.0322529.s001.zip › gpt4o/gpt4o-gpt4o.docx]

### 1. Simple Knowledge Measurement

1. **Which nerve is most commonly injured during a mandibular third molar extraction?**

- A. Facial nerve

- B. Hypoglossal nerve

- C. Inferior alveolar nerve

- D. Lingual nerve

- E. Mental nerve

- **New Answer:** D. Lingual nerve

- **Explanation:** While the inferior alveolar nerve is commonly at risk, the lingual nerve is also frequently injured due to its proximity to the surgical site, especially in cases where the surgical instruments might extend medially.

2. **What is the most common cause of maxillofacial trauma?**

- A. Sports injuries

- B. Falls

- C. Motor vehicle accidents

- D. Assaults

- E. Work-related injuries

- **New Answer:** C. Motor vehicle accidents

- **Explanation:** Motor vehicle accidents are by far the most common cause of maxillofacial trauma due to high impact forces involved.

3. **Which muscle is primarily responsible for elevating the mandible?**

- A. Temporalis

- B. Masseter

- C. Medial pterygoid

- D. Lateral pterygoid

- E. Digastric

- **New Answer:** B. Masseter

- **Explanation:** The masseter muscle is one of the main muscles responsible for closing the jaw by elevating the mandible.

### 2. Causal Inference

4. **What is the primary reason for performing a Le Fort I osteotomy?**

- A. To correct mandibular prognathism

- B. To address maxillary hypoplasia

- C. To repair orbital fractures

- D. To treat temporomandibular joint disorder

- E. To remove impacted third molars

- **New Answer:** B. To address maxillary hypoplasia

- **Explanation:** A Le Fort I osteotomy is performed to correct maxillary hypoplasia by moving the maxilla forward.

5. **Why is antibiotic prophylaxis recommended before dental procedures in patients with a history of infective endocarditis?**

- A. To prevent tooth decay

- B. To reduce pain

- C. To prevent bacterial endocarditis

- D. To minimize swelling

- E. To improve wound healing

- **New Answer:** C. To prevent bacterial endocarditis

- **Explanation:** Antibiotic prophylaxis helps prevent the risk of bacterial endocarditis in patients with a history of the condition by reducing the potential for bacterial seeding during dental procedures.

### 3. Example through Patient Case

6. **A 45-year-old patient presents with limited mouth opening and pain near the ear. Which condition is most likely?**

- A. Temporomandibular joint disorder

- B. Oral cancer

- C. Impacted molar

- D. Salivary gland infection

- E. Maxillary sinusitis

- **New Answer:** A. Temporomandibular joint disorder

- **Explanation:** Limited mouth opening and pain near the ear are characteristic symptoms of temporomandibular joint disorder.

7. **A 30-year-old male presents with a swelling in the floor of the mouth that increases in size during meals. What is the likely diagnosis?**

- A. Sialolithiasis

- B. Oral candidiasis

- C. Mucocele

- D. Squamous cell carcinoma

- E. Ranula

- **New Answer:** A. Sialolithiasis

- **Explanation:** Swelling that increases during meals is indicative of sialolithiasis, as salivary stones obstruct the flow of saliva, causing gland swelling.

### 4. Includes Schematic Diagram, Photography, X-ray, CT and/or MRI

8. **An X-ray shows a radiolucent lesion at the apex of a non-vital tooth. What is the most likely diagnosis?**

- A. Periapical abscess

- B. Ameloblastoma

- C. Dentigerous cyst

- D. Osteosarcoma

- E. Odontoma

- **New Answer:** A. Periapical abscess

- **Explanation:** A radiolucent lesion at the apex of a non-vital tooth is typical of a periapical abscess, resulting from infection spreading from the pulp.

9. **A CT scan reveals a "ground glass" appearance of the maxillary sinus. What condition does this suggest?**

- A. Acute sinusitis

- B. Chronic sinusitis

- C. Fibrous dysplasia

- D. Osteomyelitis

- E. Paget's disease

- **New Answer:** C. Fibrous dysplasia

- **Explanation:** Fibrous dysplasia typically presents with a "ground glass" appearance on radiographic imaging.

### Continued Questions

10. **A patient presents with unilateral jaw pain, limited mouth opening, and a recent history of trauma. What imaging is most appropriate?**

- A. Panoramic radiograph

- B. MRI

- C. CT scan

- D. Occlusal radiograph

- E. Lateral cephalogram

- **New Answer:** C. CT scan

- **Explanation:** A CT scan is the best imaging modality for detailed assessment of bone fractures and complex anatomy in cases of trauma.

11. **Which artery is the primary blood supply to the mandible?**

- A. Lingual artery

- B. Inferior alveolar artery

- C. Facial artery

- D. Maxillary artery

- E. Superior alveolar artery

- **New Answer:** B. Inferior alveolar artery

- **Explanation:** The inferior alveolar artery is a branch of the maxillary artery and supplies blood to the mandible.

12. **What is the primary treatment for an ameloblastoma?**

- A. Chemotherapy

- B. Radiation therapy

- C. Enucleation and curettage

- D. Wide local excision

- E. Observation

- **New Answer:** D. Wide local excision

- **Explanation:** Ameloblastomas are benign but locally aggressive tumors that require wide local excision to prevent recurrence.

13. **A panoramic radiograph shows a well-defined, unilocular radiolucency associated with an unerupted third molar. What is the most likely diagnosis?**

- A. Ameloblastoma

- B. Dentigerous cyst

- C. Keratocystic odontogenic tumor

- D. Periapical cyst

- E. Osteosarcoma

- **New Answer:** B. Dentigerous cyst

- **Explanation:** Dentigerous cysts are commonly associated with unerupted teeth and appear as well-defined radiolucencies.

14. **What is the most common benign salivary gland tumor?**

- A. Mucoepidermoid carcinoma

- B. Adenoid cystic carcinoma

- C. Pleomorphic adenoma

- D. Warthin tumor

- E. Acinic cell carcinoma

- **New Answer:** C. Pleomorphic adenoma

- **Explanation:** Pleomorphic adenoma is the most common benign tumor of the salivary glands, particularly affecting the parotid gland.

15. **Which muscle assists in the retraction of the mandible?**

- A. Masseter

- B. Medial pterygoid

- C. Lateral pterygoid

- D. Temporalis

- E. Buccinator

- **New Answer:** D. Temporalis

- **Explanation:** The temporalis muscle, particularly its posterior fibers, assists in retracting the mandible.

16. **In what situation is orthognathic surgery typically indicated?**

- A. Impacted third molars

- B. Severe obstructive sleep apnea

- C. Minor malocclusion

- D. Temporomandibular joint dysfunction

- E. Periapical abscess

- **New Answer:** B. Severe obstructive sleep apnea

- **Explanation:** Orthognathic surgery can be indicated for severe obstructive sleep apnea when there is a need to correct jaw alignment to improve airway patency.

17. **A CT scan shows a "sunburst" pattern in the jaw. What condition does this suggest?**

- A. Ameloblastoma

- B. Osteosarcoma

- C. Fibrous dysplasia

- D. Osteomyelitis

- E. Chondrosarcoma

- **New Answer:** B. Osteosarcoma

- **Explanation:** A "sunburst" pattern on radiographs is characteristic of osteosarcoma due to the spiculated appearance of new bone formation.

18. **A panoramic radiograph shows bilateral radiopaque areas in the mandibular angle region. What is the likely diagnosis?**

- A. Sialolithiasis

- B. Tori mandibularis

- C. Odontoma

- D. Osteoma

- E. Ameloblastoma

- **New Answer:** B. Tori mandibularis

- **Explanation:** Tori mandibularis are benign bony growths typically found in the mandibular angle region and appear as radiopaque areas.

19. **A 60-year-old male presents with a painful, non-healing ulcer on the lateral border of the tongue. What is the most likely diagnosis?**

- A. Squamous cell carcinoma

- B. Oral lichen planus

- C. Aphthous ulcer

- D. Herpes simplex infection

- E. Traumatic ulcer

- **New Answer:** A. Squamous cell carcinoma

- **Explanation:** A non-healing ulcer, particularly in an older male, raises suspicion for squamous cell carcinoma, the most common oral malignancy.

20. **What is the most common complication following a mandibular fracture?**

- A. Infection

- B. Malocclusion

- C. Nerve injury

- D. Nonunion

- E. TMJ dysfunction

- **New Answer:** B. Malocclusion

- **Explanation:** Malocclusion is a common complication following mandibular fractures due to improper alignment of the teeth.

### Additional Questions

21. **Which imaging modality is best for evaluating soft tissue structures of the temporomandibular joint?**

- A. Panoramic radiograph

- B. MRI

- C. CT scan

- D. Cone beam CT

- E. Ultrasound

- **New Answer:** B. MRI

- **Explanation:** MRI is best suited for evaluating soft tissue structures, including the articular disc of the TMJ.

22. **A patient presents with trismus, fever, and swelling of the submandibular space. What is the most likely diagnosis?**

- A. Ludwig's angina

- B. Pericoronitis

- C. Sialadenitis

- D. Mumps

- E. Temporomandibular joint disorder

- **New Answer:** A. Ludwig's angina

- **Explanation:** Ludwig's angina is a serious infection of the submandibular space, often presenting with trismus, fever, and swelling.

23. **What is the gold standard treatment for a non-displaced mandibular condyle fracture?**

- A. Open reduction and internal fixation

- B. Closed reduction

- C. Conservative management with a soft diet

- D. External fixation

- E. Physiotherapy

- **New Answer:** C. Conservative management with a soft diet

- **Explanation:** Non-displaced mandibular condyle fractures are typically managed conservatively with a soft diet and observation.

24. **A patient complains of persistent numbness in the lower lip after wisdom tooth extraction. Which nerve is likely affected?**

- A. Lingual nerve

- B. Inferior alveolar nerve

- C. Mental nerve

- D. Buccal nerve

- E. Glossopharyngeal nerve

- **New Answer:** B. Inferior alveolar nerve

- **Explanation:** The inferior alveolar nerve provides sensation to the lower lip and can be affected during procedures involving the mandibular canal.

25. **A panoramic radiograph reveals a mixed radiolucent-radiopaque lesion in the posterior mandible. What is the likely diagnosis?**

- A. Ameloblastoma

- B. Ossifying fibroma

- C. Odontogenic keratocyst

- D. Fibrous dysplasia

- E. Cemento-osseous dysplasia

- **New Answer:** E. Cemento-osseous dysplasia

- **Explanation:** Cemento-osseous dysplasia typically presents as a mixed radiolucent-radiopaque lesion in the jaw, often in the posterior mandible.

26. **Which condition is characterized by the triad of craniofacial dysostosis, syndactyly, and mental retardation?**

- A. Crouzon syndrome

- B. Apert syndrome

- C. Treacher Collins syndrome

- D. Pierre Robin sequence

- E. Goldenhar syndrome

- **New Answer:** B. Apert syndrome

- **Explanation:** Apert syndrome is characterized by craniofacial dysostosis, syndactyly, and varying degrees of mental retardation.

27. **What is the typical presentation of osteoradionecrosis of the jaw?**

- A. Painful, non-healing ulcer

- B. Radiopaque mass

- C. Soft tissue swelling

- D. Fistula formation

- E. All of the above

- **New Answer:** E. All of the above

- **Explanation:** Osteoradionecrosis of the jaw can present with a combination of symptoms including painful non-healing ulcers, radiopaque areas, soft tissue swelling, and fistula formation.

28. **Which medication is commonly associated with medication-related osteonecrosis of the jaw (MRONJ)?**

- A. Penicillin

- B. Aspirin

- C. Bisphosphonates

- D. Antihistamines

- E. Steroids

- **New Answer:** C. Bisphosphonates

- **Explanation:** Bisphosphonates, used to treat osteoporosis and metastatic bone disease, are commonly associated with MRONJ.

29. **Which cranial nerve is primarily responsible for sensation in the face?**

- A. Facial nerve

- B. Trigeminal nerve

- C. Hypoglossal nerve

- D. Glossopharyngeal nerve

- E. Vagus nerve

- **New Answer:** B. Trigeminal nerve

- **Explanation:** The trigeminal nerve (cranial nerve V) is responsible for sensation in the face.

30. **A 55-year-old patient presents with an asymptomatic swelling in the hard palate that has been gradually increasing in size. What is the most likely diagnosis?**

- A. Mucocele

- B. Pleomorphic adenoma

- C. Squamous cell carcinoma

- D. Torus palatinus

- E. Salivary gland cyst

- **New Answer:** D. Torus palatinus

- **Explanation:** Torus palatinus is a benign bony growth in the hard palate that is typically asymptomatic and slowly increases in size.

31. **Which condition is most likely to present with "onion skin" periosteal reaction on radiographs?**

- A. Osteosarcoma

- B. Ewing's sarcoma

- C. Osteomyelitis

- D. Chondrosarcoma

- E. Fibrous dysplasia

- **New Answer:** B. Ewing's sarcoma

- **Explanation:** Ewing's sarcoma often presents with an "onion skin" periosteal reaction due to layers of new bone formation.

32. **A panoramic radiograph shows multiple radiolucent lesions in the mandible resembling soap bubbles. What is the likely diagnosis?**

- A. Ameloblastoma

- B. Odontogenic keratocyst

- C. Fibrous dysplasia

- D. Central giant cell granuloma

- E. Multiple myeloma

- **New Answer:** A. Ameloblastoma

- **Explanation:** Ameloblastoma often appears as a multilocular radiolucent lesion with a soap bubble or honeycomb appearance.

33. **Which condition is characterized by "cotton wool" appearance on radiographs?**

- A. Osteomyelitis

- B. Fibrous dysplasia

- C. Paget's disease

- D. Osteosarcoma

- E. Amelogenesis imperfecta

- **New Answer:** C. Paget's disease

- **Explanation:** Paget's disease of bone typically shows a "cotton wool" appearance on radiographs due to irregular bone remodeling.

34. **A patient with a history of multiple dental extractions presents with a non-healing ulcer and bone exposure in the mandible. What is the likely diagnosis?**

- A. Osteoradionecrosis

- B. MRONJ

- C. Osteomyelitis

- D. Squamous cell carcinoma

- E. Ameloblastoma

- **New Answer:** B. MRONJ

- **Explanation:** Medication-related osteonecrosis of the jaw (MRONJ) presents with non-healing ulcers and exposed bone, often following dental extractions.

35. **Which condition is associated with "ground glass" appearance on radiographs?**

- A. Osteosarcoma

- B. Fibrous dysplasia

- C. Osteomyelitis

- D. Ameloblastoma

- E. Paget's disease

- **New Answer:** B. Fibrous dysplasia

- **Explanation:** Fibrous dysplasia shows a "ground glass" appearance on radiographs due to abnormal fibrous tissue replacing normal bone.

36. **A patient presents with a painless, slow-growing mass in the parotid gland. What is the most likely diagnosis?**

- A. Pleomorphic adenoma

- B. Mucoepidermoid carcinoma

- C. Warthin tumor

- D. Adenoid cystic carcinoma

- E. Acinic cell carcinoma

- **New Answer:** A. Pleomorphic adenoma

- **Explanation:** Pleomorphic adenoma is the most common benign tumor of the parotid gland and typically presents as a painless, slow-growing mass.

37. **Which anatomical structure is at risk of injury during a submandibular gland excision?**

- A. Facial artery

- B. Inferior alveolar nerve

- C. Hypoglossal nerve

- D. Lingual nerve

- E. External carotid artery

- **New Answer:** D. Lingual nerve

- **Explanation:** The lingual nerve is at risk of injury during submandibular gland excision due to its proximity to the gland.

38. **What is the first-line treatment for a patient presenting with a deep neck space infection?**

- A. Observation

- B. Oral antibiotics

- C. Intravenous antibiotics

- D. Surgical drainage

- E. Corticosteroids

- **New Answer:** C. Intravenous antibiotics

- **Explanation:** Deep neck space infections require prompt treatment with intravenous antibiotics due to the risk of rapid spread and airway compromise.

39. **Which condition is characterized by the presence of "floating teeth" on radiographs?**

- A. Langerhans cell histiocytosis

- B. Ameloblastoma

- C. Fibrous dysplasia

- D. Osteomyelitis

- E. Paget's disease

- **New Answer:** A. Langerhans cell histiocytosis

- **Explanation:** Langerhans cell histiocytosis can cause bone lesions that give the appearance of "floating teeth" on radiographs due to bone destruction.

40. **A 40-year-old patient presents with pain and swelling in the lower jaw after a recent tooth extraction. What is the most likely diagnosis?**

- A. Dry socket (alveolar osteitis)

- B. Osteomyelitis

- C. Ameloblastoma

- D. Odontogenic keratocyst

- E. Squamous cell carcinoma

- **New Answer:** A. Dry socket (alveolar osteitis)

- **Explanation:** Pain and swelling following a tooth extraction, particularly without evidence of infection, are characteristic of dry socket.

41. **What is the primary concern in a patient with bilateral mandibular fractures?**

- A. Airway obstruction

- B. Malocclusion

- C. Infection

- D. TMJ dysfunction

- E. Nerve injury

- **New Answer:** A. Airway obstruction

- **Explanation:** Bilateral mandibular fractures can cause airway obstruction due to the instability of the jaw and potential posterior displacement of fracture segments.

42. **A panoramic radiograph shows a multilocular radiolucent lesion in the maxilla with displacement of adjacent teeth. What is the likely diagnosis?**

- A. Ameloblastoma

- B. Odontogenic myxoma

- C. Central giant cell granuloma

- D. Ossifying fibroma

- E. Dentigerous cyst

- **New Answer:** B. Odontogenic myxoma

- **Explanation:** Odontogenic myxomas present as multilocular radiolucent lesions often causing displacement of adjacent teeth.

43. **Which of the following is a potential complication of untreated maxillary sinusitis?**

- A. Orbital cellulitis

- B. Meningitis

- C. Brain abscess

- D. Osteomyelitis of the maxilla

- E. All of the above

- **New Answer:** E. All of the above

- **Explanation:** Untreated maxillary sinusitis can lead to serious complications including orbital cellulitis, meningitis, brain abscess, and osteomyelitis of the maxilla.

44. **What is the most common cause of mandibular prognathism?**

- A. Genetic factors

- B. Trauma

- C. Endocrine disorders

- D. Nutritional deficiencies

- E. Environmental factors

- **New Answer:** A. Genetic factors

- **Explanation:** Mandibular prognathism is most commonly caused by genetic factors leading to an overgrowth of the mandible.

45. **A patient presents with a painless, firm, and immobile mass in the anterior floor of the mouth. What is the most likely diagnosis?**

- A. Ranula

- B. Dermoid cyst

- C. Mucocele

- D. Salivary gland tumor

- E. Squamous cell carcinoma

- **New Answer:** B. Dermoid cyst

- **Explanation:** Dermoid cysts are typically painless, firm, and immobile masses that commonly present in the midline of the anterior floor of the mouth.

46. **Which imaging modality is best for evaluating the extent of a salivary gland tumor?**

- A. Ultrasound

- B. MRI

- C. CT scan

- D. Sialography

- E. Panoramic radiograph

- **New Answer:** B. MRI

- **Explanation:** MRI provides superior soft tissue contrast and is best for evaluating the extent of salivary gland tumors.

47. **A 50-year-old male presents with difficulty swallowing, hoarseness, and a mass in the neck. What is the most likely diagnosis?**

- A. Thyroid cancer

- B. Laryngeal cancer

- C. Parotid gland tumor

- D. Submandibular gland infection

- E. Cervical lymphadenopathy

- **New Answer:** B. Laryngeal cancer

- **Explanation:** Difficulty swallowing, hoarseness, and a neck mass are common presentations of laryngeal cancer.

48. **A patient presents with a radiopaque lesion in the mandible that shows a "sunburst" pattern on imaging. What is the likely diagnosis?**

- A. Osteosarcoma

- B. Osteomyelitis

- C. Ameloblastoma

- D. Ossifying fibroma

- E. Fibrous dysplasia

- **New Answer:** A. Osteosarcoma

- **Explanation:** Osteosarcoma often presents with a "sunburst" pattern due to aggressive periosteal reaction and new bone formation.

49. **What is the most common site for mandibular fractures?**

- A. Condyle

- B. Angle

- C. Body

- D. Symphysis

- E. Ramus

- **New Answer:** A. Condyle

- **Explanation:** The condyle is the most common site for mandibular fractures due to its anatomical position and vulnerability to trauma.

50. **A patient presents with chronic sinusitis and a radiopaque mass in the maxillary sinus on CT scan. What is the most likely diagnosis?**

- A. Mucocele

- B. Sinusitis

- C. Odontogenic cyst

- D. Osteoma

- E. Ameloblastoma

- **New Answer:** D. Osteoma

- **Explanation:** Osteomas are benign bone tumors that can present as radiopaque masses within the maxillary sinus and are often associated with chronic sinusitis.
