# Supplementary material for: How valuable are the questions and answers generated by large language models in oral and maxillofacial surgery?
Source: PLoS One. 2025 May 28;20(5):e0322529. doi: 10.1371/journal.pone.0322529 (PMC12119001; doi:10.1371/journal.pone.0322529)
Supplement: Attachment — Submitted filename: Response to Reviewers_21Feb2025.docx [file pone.0322529.s004.docx]

Reviewer #2:

Thank you for addressing the reviewers comments which resulted in the improvement of the manuscript.

>> Thank you very much. Your reviews have greatly improved this manuscript.

Reviewer #5:

As I said in the first review, the work of is of great value. It is well done work. The few minor comments that I raised were not done. There is also no response to the reviewers' comments.

>> Following your comments, we have revisited your comments raised in the previous round.

Seeing this groundbreaking and superb work that links the application of cutting-edge IT technologies for human health excites me greatly. It's a very outstanding piece of scientific work. I honestly believe that it deserves to be published.
The research was done in a really good manner. The introduction, abstract, and discussion sections are all greatly interesting.
I only have a few small things to say.
You may review the font size, spacing, and typography of the whole text and make revisions in accordance with the journal's guidelines.
>> I think this was revised in the previous round.

It is understandable that the research did not involve human subjects. The following topics in the methodology and materials section, however, require further clarification.

>> For these comments, I revised the methods as follows:

= When and where was the study carried out?

This study evaluated three LLMs: ChatGPT4, ChatGPT4o, and Claude3-Opus. The models were tested using updates from May 16, 2024 (ChatGPT4 and ChatGPT4o), and February 06, 2024 (Claude3-Opus). This study was conducted between June 12, 2024, and July 10, 2024, at Wonkwang and Gachon University, Republic of Korea.

= How was the analysis carried out?

**Question Generation and Evaluation**

Three LLMs were prompted with the following request (Fig. 1):

Create 50 questions for the Oral and Maxillofacial Surgery Specialist Exam to find 1 correct answer out of 5 options and provide the correct answer for each question and the basis for the correct answer.

Each question must meet one or more of the following:

1. Simple knowledge measurement

2. Causal inference

3. Example through patient case

4. Includes schematic diagram, photography, X-ray, CT and/or MRI

**Image Integration and Modification**

For questions requiring images, open-source images from Google were incorporated, ensuring they were sourced only from credible institutions. Image utilization per model was as follows: ChatGPT-4 (9 questions), ChatGPT-4o (18 questions), and Claude 3-Opus (18 questions). When only a specific part of an image needed to be evaluated, relevant areas were highlighted using arrows or circles for clarity (Fig. 2). To assess LLMs' ability to generate independent explanations, the provided answers and rationales were removed before resubmitting the questions to the models.

= Measurements, experimental procedures,

**Performance Evaluation and Statistical Analysis**

The LLMs' capabilities were assessed based on:

- Logical Accuracy and Thoroughness: If an explanation contained correct isolated facts but lacked logical relevance to the problem, it was deemed incorrect. Similarly, a correct solution with an explanation consisting only of disjointed facts was considered insufficient.
- Quantitative Assessment: Responses were classified as correct or incorrect, and explanations were labeled as detailed or insufficient. Image interpretation abilities were also evaluated, and cross-verifications were performed to ensure logical consistency.
- Reliability Testing: Intra-rater and inter-rater reliability were assessed for all 150 questions. Each expert evaluated all questions twice at a one-week interval, and a final gold-standard evaluation was established based on consensus.
- Statistical Analysis: An independent t-test was conducted to compare performance metrics among the three LLMs. Accuracy, precision, recall, and F1-scores were calculated using Scikit-learn version 1.4.2. The statistical analyses assumed normality in data distribution and independence between observations. Adjustments for multiple comparisons were considered unnecessary as the analyses were hypothesis-driven and controlled.

= Who were the specialists who assessed the 150 questions that were produced? How did they get chosen?
**Expert Review Process**

Two oral and maxillofacial surgeons assessed the generated questions. These specialists were selected based on their expertise in oral and maxillofacial surgery and their prior experience with standardized examination question development. The review process involved:

- Verifying compliance with predefined criteria (knowledge measurement, causal inference, patient case-based scenarios, and image inclusion).
- Assessing clinical relevance, alignment with standardized exam formats, and logical coherence of the provided explanations.
- Evaluating the accuracy of answers, completeness of problem-solving approaches, and consistency of logical reasoning.

Categorizing questions into subfields such as dentoalveolar, trauma, infection, and cysts.

The conclusion is extremely short. The conclusions section should be well-written and provide closure because it is the last section in which the findings of this great work are summed up.

>> I think this was revised in the previous round.
