# Supplementary material for: How valuable are the questions and answers generated by large language models in oral and maxillofacial surgery?
Source: PLoS One. 2025 May 28;20(5):e0322529. doi: 10.1371/journal.pone.0322529 (PMC12119001; doi:10.1371/journal.pone.0322529)
Supplement: Attachment — Submitted filename: Response to Reviewers_02Mar2025.docx [file pone.0322529.s005.docx]

Seeing this groundbreaking and superb work that links the application of cutting-edge IT technologies for human health excites me greatly. It's a very outstanding piece of scientific work. I honestly believe that it deserves to be published.
The research was done in a really good manner. The introduction, abstract, and discussion sections are all greatly interesting.
I only have a few small things to say.

You may review the font size, spacing, and typography of the whole text and make revisions in accordance with the journal's guidelines.

>> Based on your feedback, we have revised the font size, spacing, and typography of the whole text in accordance with the journal's guidelines.

It is understandable that the research did not involve human subjects. The following topics in the methodology and materials section, however, require further clarification.
>> To clarify the materials and methods, we added the following figure.

Figure 1. A flow diagram showing the process by which large language models generate and answer questions about oral and maxillofacial surgery in this study. LLM: large language models, OMFS: oral and maxillofacial surgeon.

Figure 2. An example of inserting images verified and labeled by experts from credible institutions among open sources into questions generated by large language models.

= When and where was the study carried out?

>> This study was conducted between June 12, 2024, and July 10, 2024, at Wonkwang and Gachon University, Republic of Korea. We revised the methods as follows:

This study evaluated three LLMs: ChatGPT4, ChatGPT4o, and Claude3-Opus. The models were tested using updates from May 16, 2024 (ChatGPT4 and ChatGPT4o), and February 06, 2024 (Claude3-Opus). This study was conducted between June 12, 2024, and July 10, 2024, at Wonkwang and Gachon University, Republic of Korea.

= How was the analysis carried out?

>> We conducted Performance Evaluation and Statistical Analysis as follows:

The LLMs' capabilities were assessed based on:

 Logical Accuracy and Thoroughness: If an explanation contained correct isolated facts but lacked logical relevance to the problem, it was deemed incorrect. Similarly, a correct solution with an explanation consisting only of disjointed facts was considered insufficient.

 Quantitative Assessment: Responses were classified as correct or incorrect, and explanations were labeled as detailed or insufficient. Image interpretation abilities were also evaluated, and cross-verifications were performed to ensure logical consistency.

 Reliability Testing: Intra-rater and inter-rater reliability were assessed for all 150 questions. Each expert evaluated all questions twice at a one-week interval, and a final gold-standard evaluation was established based on consensus.

 Statistical Analysis: An independent t-test was conducted to compare performance metrics among the three LLMs. Accuracy, precision, recall, and F1-scores were calculated using Scikit-learn version 1.4.2. The statistical analyses assumed normality in data distribution and independence between observations. Adjustments for multiple comparisons were considered unnecessary as the analyses were hypothesis-driven and controlled.

= Measurements, experimental procedures,

>> We had LLMs generate questions and evaluated the questions they generated. For questions that require an image, we found and inserted the appropriate image. And each LLM was asked to solve the questions and explain them appropriately. We revised the methods as follows:

**Question Generation and Evaluation**

Three LLMs were prompted with the following request (Fig. 1):

Create 50 questions for the Oral and Maxillofacial Surgery Specialist Exam to find 1 correct answer out of 5 options and provide the correct answer for each question and the basis for the correct answer.

Each question must meet one or more of the following:

1. Simple knowledge measurement

2. Causal inference

3. Example through patient case

4. Includes schematic diagram, photography, X-ray, CT and/or MRI

**Expert Review Process**

Two oral and maxillofacial surgeons assessed the generated questions. These specialists were selected based on their expertise in oral and maxillofacial surgery and their prior experience with standardized examination question development. The review process involved:

 Verifying compliance with predefined criteria (knowledge measurement, causal inference, patient case-based scenarios, and image inclusion).

 Assessing clinical relevance, alignment with standardized exam formats, and logical coherence of the provided explanations.

 Evaluating the accuracy of answers, completeness of problem-solving approaches, and consistency of logical reasoning.

 Categorizing questions into subfields such as dentoalveolar, trauma, infection, and cysts.

**Image Integration and Modification**

For questions requiring images, open-source images from Google were incorporated, ensuring they were sourced only from credible institutions. Image utilization per model was as follows: ChatGPT-4 (9 questions), ChatGPT-4o (18 questions), and Claude 3-Opus (18 questions). When only a specific part of an image needed to be evaluated, relevant areas were highlighted using arrows or circles for clarity (Fig. 2). To assess LLMs' ability to generate independent explanations, the provided answers and rationales were removed before resubmitting the questions to the models.

= Who were the specialists who assessed the 150 questions that were produced? How did they get chosen?
>> The specialists who assessed the 150 questions that were produced were two oral and maxillofacial surgeons. These specialists were selected based on their expertise in oral and maxillofacial surgery and their prior experience with standardized examination question development. We revised the methods as follows:

**Expert Review Process**

Two oral and maxillofacial surgeons assessed the generated questions. These specialists were selected based on their expertise in oral and maxillofacial surgery and their prior experience with standardized examination question development. The review process involved:

 Verifying compliance with predefined criteria (knowledge measurement, causal inference, patient case-based scenarios, and image inclusion).

 Assessing clinical relevance, alignment with standardized exam formats, and logical coherence of the provided explanations.

 Evaluating the accuracy of answers, completeness of problem-solving approaches, and consistency of logical reasoning.

 Categorizing questions into subfields such as dentoalveolar, trauma, infection, and cysts.

The conclusion is extremely short. The conclusions section should be well-written and provide closure because it is the last section in which the findings of this great work are summed up.

>> Based on your comments, we have revised the conclusion as follows:

This study demonstrates that while LLMs like ChatGPT4, ChatGPT4o, and Claude3-Opus exhibit robust capabilities in generating and solving oral and maxillofacial surgery questions, their performance is not without limitations. None of the models were able to answer correctly all the questions they generated themselves, highlighting persistent challenges such as AI hallucinations and contextual understanding gaps. The results also emphasize the importance of multimodal inputs, as questions with annotated images achieved higher accuracy rates compared to text-only prompts. Despite these shortcomings, the LLMs showed significant promise in problem-solving, logical consistency, and response fidelity, particularly in structured or numerical contexts. Future research should focus on refining these models through HITL approaches, expanding sample sizes, and exploring their adaptability to dynamic medical knowledge and ethical considerations in clinical practice.
